# Collagen IV of basement membranes: III. Chloride pressure is a primordial innovation that drives and maintains the assembly of scaffolds

**DOI:** 10.1016/j.jbc.2023.105318

**Published:** 2023-10-04

**Authors:** Sergei P. Boudko, Aspirnauts, Octavia Ailsworth, ZaKylah Bryant, Camryn Cole, Jacob Edward, Di’Andra Edwards, Sydney Farrar, Julianna Gallup, Michael Gallup, Martina Gergis, Aalia Holt, Madeline Lach, Elizabeth Leaf, Finn Mahoney, Max McFarlin, Monica Moran, Galeesa Murphy, Charlotte Myers, Connie Ni, Neve Redhair, Rocio Rosa, Olivia Servidio, Jaeden Sockbeson, Lauren Taylor, Vadim K. Pedchenko, Elena N. Pokidysheva, Alena M. Budko, Rachel Baugh, Patrick Toby Coates, Aaron L. Fidler, Heather M. Hudson, Sergey V. Ivanov, Carl Luer, Tetyana Pedchenko, Robert L. Preston, Mohamed Rafi, Roberto Vanacore, Gautam Bhave, Julie K. Hudson, Billy G. Hudson

**Affiliations:** 1 Division of Nephrology and Hypertension, Department of Medicine, Vanderbilt University Medical Center, Nashville, Tennessee, USA; 2 Center for Matrix Biology, Vanderbilt University Medical Center, Nashville, Tennessee, USA; 3 Department of Biochemistry, Vanderbilt University, Nashville, Tennessee, USA; 4 Oregon Charter Academy, Mill City, Oregon, USA; 5 Department of Medical Education and Administration, Vanderbilt University Medical Center, Nashville, Tennessee, USA; 6 Central Northern Adelaide Renal and Transplantation Service, Royal Adelaide Hospital, Adelaide, Australia; 7 Department of Rehabilitation Medicine, University of Kansas Medical Center, Kansas City, Kansas, USA; 8 Mote Marine Laboratory, Sarasota, Florida, USA; 9 School of Biological Sciences, Illinois State University, Normal, Illinois, USA; 10 Vanderbilt-Ingram Cancer Center, Vanderbilt University, Nashville, Tennessee, USA; 11 Vanderbilt Institute of Chemical Biology, Vanderbilt University, Nashville, Tennessee, USA; 12 Department of Biological Sciences, Vanderbilt University, Nashville, Tennessee, USA; 13 Department of Cell and Developmental Biology, Vanderbilt University, Nashville, Tennessee, USA; 14 Department of Pathology, Microbiology, and Immunology, Vanderbilt University Medical Center, Nashville, Tennessee, USA

**Keywords:** collagen IV, basement membrane, NC1 domain, chloride, protein self-assembly, protein stability, protein evolution, extracellular matrix, phylogeny, small molecule

## Abstract

Collagen IV scaffold is a primordial innovation enabling the assembly of a fundamental architectural unit of epithelial tissues—a basement membrane attached to polarized cells. A family of six α-chains (α1 to α6) coassemble into three distinct protomers that form supramolecular scaffolds, noted as collagen IV^α121^, collagen IV^α345^, and collagen IV^α121–α556^. Chloride ions play a pivotal role in scaffold assembly, based on studies of NC1 hexamers from mammalian tissues. First, Cl^−^ activates a molecular switch within trimeric NC1 domains that initiates protomer oligomerization, forming an NC1 hexamer between adjoining protomers. Second, Cl^−^ stabilizes the hexamer structure. Whether this Cl^−^-dependent mechanism is of fundamental importance in animal evolution is unknown. Here, we developed a simple *in vitro* method of SDS-PAGE to determine the role of solution Cl^−^ in hexamer stability. Hexamers were characterized from 34 animal species across 15 major phyla, including the basal Cnidarian and Ctenophora phyla. We found that solution Cl^−^ stabilized the quaternary hexamer structure across all phyla except Ctenophora, Ecdysozoa, and Rotifera. Further analysis of hexamers from peroxidasin knockout mice, a model for decreasing hexamer crosslinks, showed that solution Cl^−^ also stabilized the hexamer surface conformation. The presence of sufficient chloride concentration in solution or “chloride pressure” dynamically maintains the native form of the hexamer. Collectively, our findings revealed that chloride pressure on the outside of cells is a primordial innovation that drives and maintains the quaternary and conformational structure of NC1 hexamers of collagen IV scaffolds.

Collagen IV scaffold is a primordial innovation enabling the assembly of a fundamental architectural unit of epithelial tissues—a basement membrane (BM) attached to polarized cells (1, 2, 3). Experimental disruption of scaffolds causes BM destabilization and tissue dysfunction in mice, zebrafish, flies, and nematodes (3, 4, 5, 6, 7, 8). Moreover, genetic defects cause Alport syndrome, Gould syndrome, and HANAC in humans affecting millions worldwide. In mammals, a family of six collagen α-chains (α1 to α6) coassemble into three distinct supramolecular scaffolds, noted as collagen IV^α121^, collagen IV^α345^, and collagen IV^α121–α556^ (9, 10, 11, 12, 13, 14) as reviewed in an accompanying article. The collagen IV^α121^ scaffold is ubiquitously expressed in tissues and organs, whereas the others have restricted distributions.

Chloride ions play a pivotal role in scaffold assembly (15, 16, 17, 18, 19, 20). Scaffold formation begins with the trimerization of α-chains into triple helical protomers inside the cell. Protomers are then secreted and undergo oligomerization forming a supramolecular scaffold outside the cell. Group 1 Cl^−^ activates a molecular switch within NC1 domains that initiates oligomerization of two protomers forming an NC1 hexamer at the trimer–trimer interface (Fig. 1*A*) (15, 17, 18). In turn, group 2 Cl^−^ dynamically stabilizes and maintains the quaternary hexamer structure (Fig. 1*A*) (17, 18). Altogether, 12 Cl^−^ ions belonging to groups 1 and 2 form a ring at the interface (Fig. S1). Then covalent sulfilimine crosslinks reinforce the stability of quaternary structure (Fig. 1*A*) (21, 22). Subsequently, four protomers oligomerize *via* the 7S domain forming a tetrameric structure in the final step of scaffold assembly (Fig. 1*B*) (23, 24, 25).

**Figure 1:**
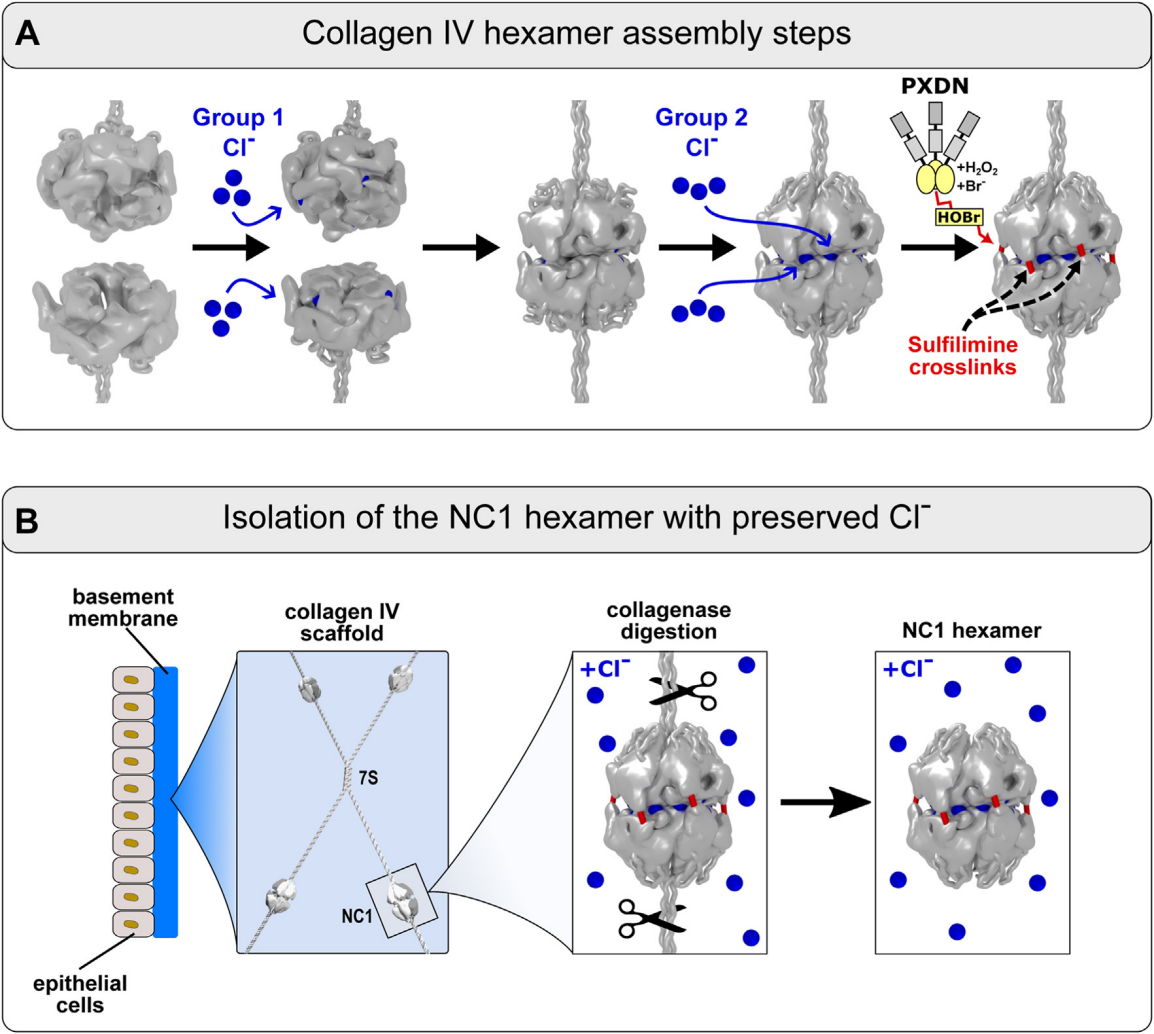
**Role of chloride in assembly and maintenance of collagen IV hexamer in mammals.***A*, chloride triggers the collagen IV hexamer assembly and dynamically stabilizes the structure until the NC1 hexamer gets reinforced by covalent sulfilimine crosslinks. Six Cl^−^ ions of group 1 get incorporated into NC1 trimers and modify the interacting surface, making it compatible with the hexamer assembly (15, 16). Once a transient hexamer is formed, it gets stabilized by six Cl^−^ ions of group 2. Overall, 12 Cl^−^ ions are incorporated at the hexamer interface. Until the hexamer is not reinforced by sulfilimine bonds, it requires the presence of physiologically normal Cl^−^ concentration in solution (∼100 mM) or higher to maintain the intact hexamer structure (17, 18, 20). Once covalently crosslinked by sulfilimine bonds, the hexamer becomes protected from dissociation upon depletion of Cl^−^ from solution. *B*, collagen IV scaffold is the core of every functional basement membrane. The 7S dodecamer assembly of the N termini of four protomers together with the NC1 hexamer complex formed at the C termini are two major building blocks of the continuous collagen IV scaffold. The role of chloride ions in maintaining the integrity of the NC1 hexamer in other animals was the subject of this study. To investigate the role of chloride, all sample preparations were done in the presence of 150 mM NaCl to preserve Cl^−^ before the analysis. The NC1 hexamer was liberated from insoluble tissue material by collagenase digestion.

This Cl^−^-dependent mechanism is based on extensive studies of the chemical and physical properties of NC1 hexamers that were isolated from bovine and human tissues (Fig. 1*B*) (15). Notably, crystal structures of α121 and α345 hexamers show the atomic location of a ring of 12 Cl^−^, composed of two groups of six ions each, at the trimer–trimer interface (17, 18, 19, 26). Alignment of amino acid sequences of NC1 domains across several metazoans indicated that Cl^−^-binding sites are conserved across bilaterians and nonbilaterians, with a few exceptions such as fruit fly and comb jelly (Fig. S2) (12). The conservation of binding sites suggests that Cl^−^ ions play a fundamental role in the assembly of collagen IV scaffolds across the animal kingdom.

Knowledge of the evolutionary role of Cl^−^ ions in assembly may shed light on functionality of collagen IV scaffolds in the genesis of tissues and organs and dysfunction in disease. Here, we developed a simple *in vitro* method of SDS-PAGE to determine the role of Cl^−^ in hexamer stability. The methodology was amenable for a cohort of high-school and undergraduate students to participate in an expedition on Cl^−^ function to the dawn of the animal kingdom. Collectively, their findings reflect a primordial function of chloride pressure that drives and maintains the assembly of collagen IV scaffolds.

## Results

### Development of an SDS-PAGE method for analysis of Cl^−^ impact on hexamer stability

In previous studies, we used the size-exclusion chromatography (SEC) under nondenaturing conditions to ascertain whether Cl^−^ stabilizes hexamer quaternary structure in bovine and human tissues. We found that depletion of Cl^−^ induced the dissociation of lens capsule BM (LBM) α121 hexamers (15) and recombinant α345 hexamer (20), devoid of crosslinks, into monomer subunits. These findings revealed that Cl^−^ stabilizes the hexamer quaternary structure. In contrast, crosslinked hexamers were found resistant to dissociation and, therefore not amenable for study by SEC.

**Figure 2:**
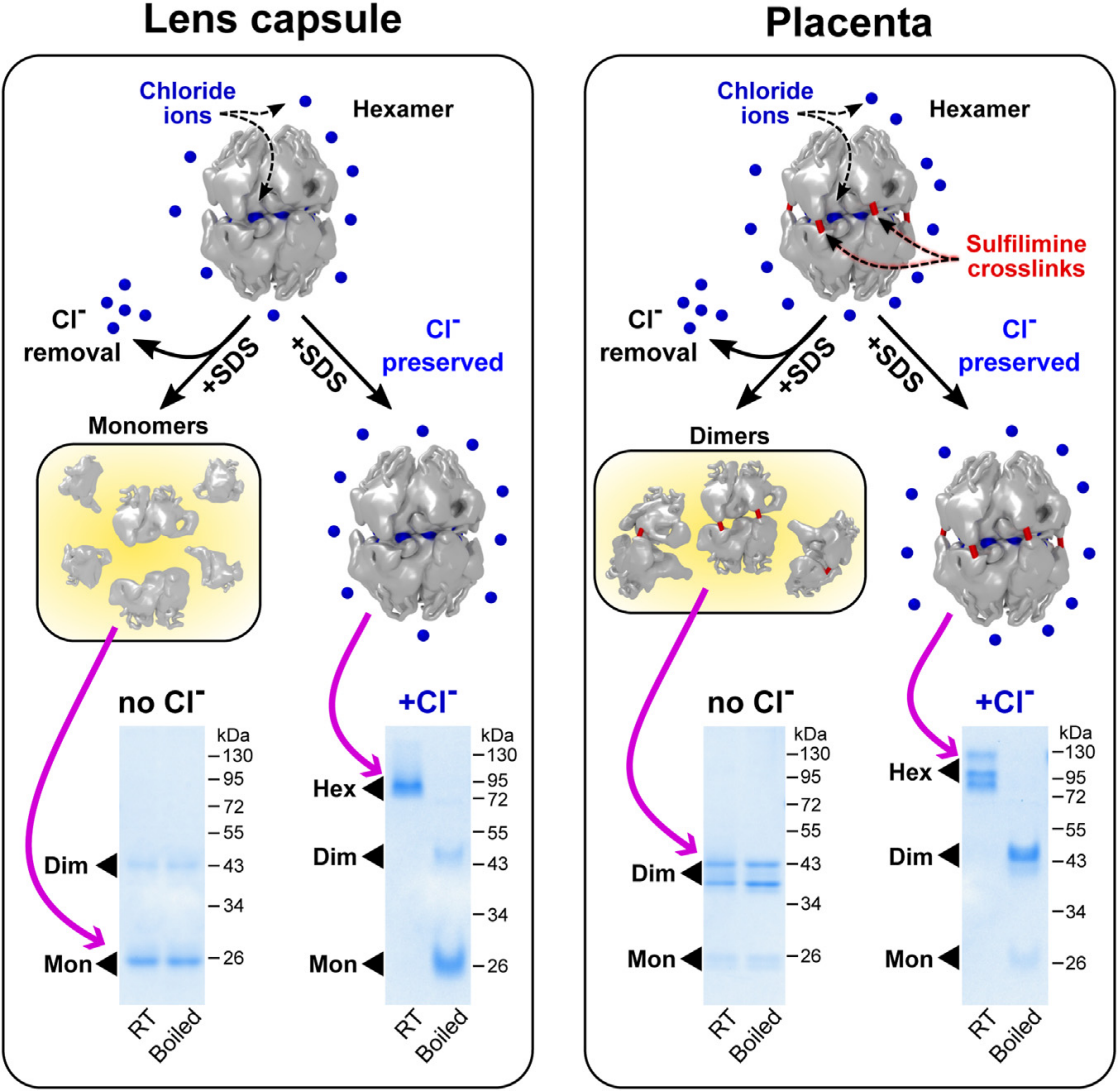
**Hexamer stability can be challenged by SDS in the presence or the absence of sufficient chloride concentration.** Regular SDS-PAGE analysis of the NC1 hexamers purified from lens capsule BM (LBM, low amount of sulfilimine crosslinks) and placenta (PBM, high amount of sulfilimine crosslinks) shows no signs of SDS-resistant hexamer. Regardless of sample boiling, the hexamer dissociates into dimers (sulfilimine crosslinked) and monomers. The presence of chloride protects the hexamers from dissociation by SDS at room temperature. Indeed, both LBM and PBM NC1 hexamers demonstrate SDS resistance in the presence of Cl^−^ at room temperature. Upon boiling, the hexamers dissociate into dimers and monomers and serve as controls. BM, basement membrane; Dim, dimer; Hex, hexamer; Mon, monomer; RT, room temperature.

Given that crosslinked hexamers predominate in animal tissues, we developed a simple *in vitro* method to measure Cl^−^ impact on hexamer stability using SDS-PAGE. Serendipitously, we found that the native hexamer is stable at room temperature in 1% SDS, a protein denaturant, and in the presence of 150 mM Cl^−^. However, in the absence of Cl^−^, the hexamer dissociates into subunits. These observations provided a strategy to modify the traditional SDS-PAGE into a novel method for analysis of Cl^−^ impact. Hexamers were excised from tissues by collagenase digestion in the presence of 150 mM Cl^−^ to preserve the native structure harboring the Cl^−^ ions (Fig. 1*B*). Samples were prepared for electrophoresis with or without Cl^−^ and at room temperature or boiled just before starting the electrophoresis in the standard fashion. Boiled samples served as a positive control for fully dissociated subunits. Accordingly, Cl^−^ was included or excluded from the gel and the running buffer. To demonstrate utility of this method, we analyzed uncrosslinked hexamer from LBM (Fig. 2, *left panel*). A comparison of gel patterns of samples prepared at room temperature, with and without Cl^−^, revealed that Cl^−^ stabilized the quaternary hexamer structure at room temperature. This finding is analogous to our previous study using SEC analysis of LBM hexamer under nondenaturing conditions (15). We repeated the SDS-PAGE analysis on crosslinked placenta (placenta BM) hexamer (Fig. 2, *right panel*), which showed that Cl^−^ stabilized the hexamer and prevented dissociation into dimer subunits. Collectively, the results demonstrated the capacity of the SDS-PAGE method to distinguish the impact of Cl^−^ on hexamer stability in the context of crosslinked hexamers.

This simple assay could be applicable in studies of other small-molecule–dependent proteins and complexes. To our knowledge, no proteins have been reported that demonstrate SDS resistance with the requirement of supplementing specific solutes. It was previously shown that SDS resistance is a common property of kinetically stable proteins with a bias toward a very high content β-sheet structure (27). In our case, proteins with very similar fold and secondary structure content (relatively high content of β-sheet structure) demonstrated two extreme cases of SDS resistance requiring or not the presence of chloride in solution.

### Cl^−^ pressure stabilizes the quaternary structure of hexamer across animalia

The simple *in vitro* method of SDS-PAGE provided a strategy for a cohort of 23 high-school and undergraduate students to participate on an expedition to determine whether Cl^−^ plays a role in the maintenance of hexamer structure in diverse animals ranging from humans to Ctenophores. In support of their research activities, students were mentored in professional development, self-discovery, and wellness (supporting information 2). Of note, the students were from disadvantaged and diverse backgrounds.

**Figure 3:**
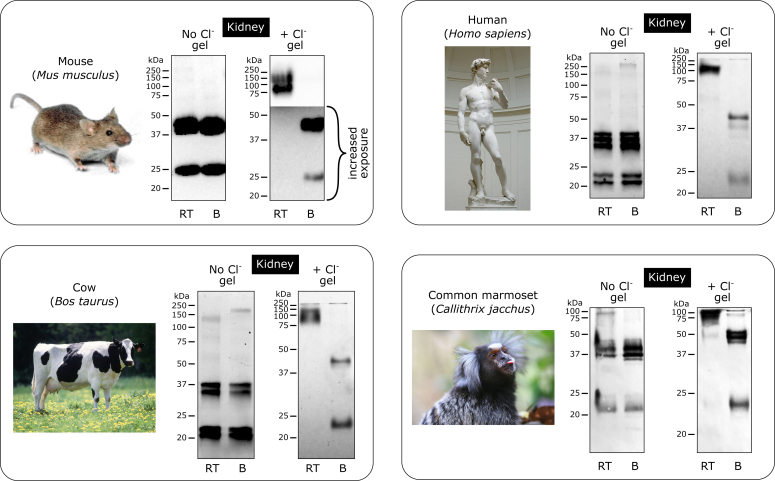
**Chloride pressure is required to stabilize the NC1 hexamer in Mammalia.** All species demonstrated a requirement of chloride supplement to stabilize the NC1 hexamer. Animal images from Wikimedia Commons (commons.wikimedia.org) are used for illustration purposes only. B, boiled sample; RT, room temperature sample.

**Figure 4:**
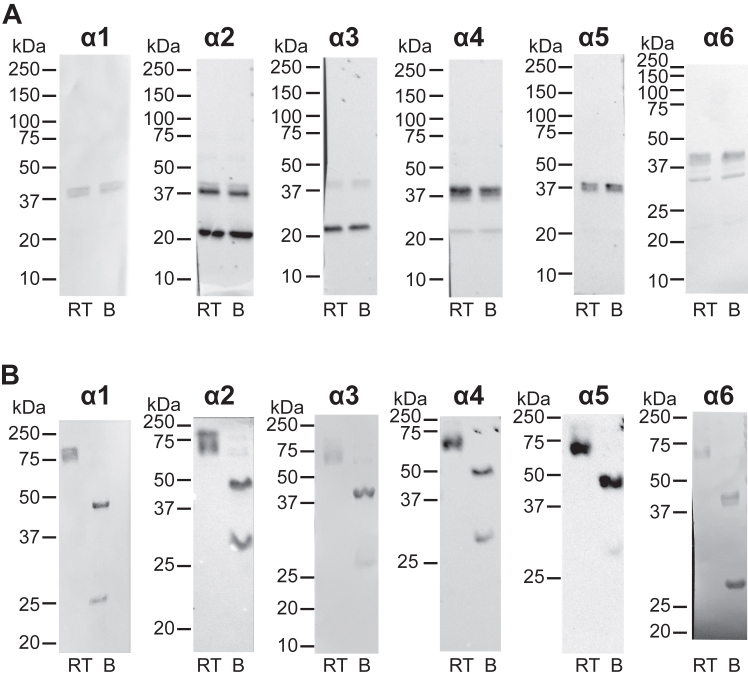
**Chloride pressure is required to stabilize all isoforms of mammalian collagen IV hexamers.** In mammals, six α chains of collagen IV form trimeric protomers of the following compositions: α121, α345, and α565, which associate into hexamers α121/α121, α345/α345, and α121/α565. The NC1 hexamers isolated from bovine kidney were used to investigate chain-specific chloride dependency for hexamer stability. *A*, Western blots with chain-specific antibodies performed on nonboiled (room temperature [RT]) and boiled (B) samples separated on regular SDS-PAGE without Cl^−^ supplement. *B*, Western blots for the samples separated on SDS-PAGE supplemented with 150 mM NaCl. Only in the presence of chloride supplement, each chain was found in the hexamer, whereas in the absence of chloride, both nonheated and heated samples ran as dimers and monomers. Thus, all known hexamer isoforms of collagen IV require chloride supplement for stability. The α-chain-specific antibodies used were H11 (α1 specific), H22 (α2 specific), Mab3 (α3 specific), H43 (α4 specific), H52 (α5 specific), and B66 (α6 specific).

**Figure 5:**
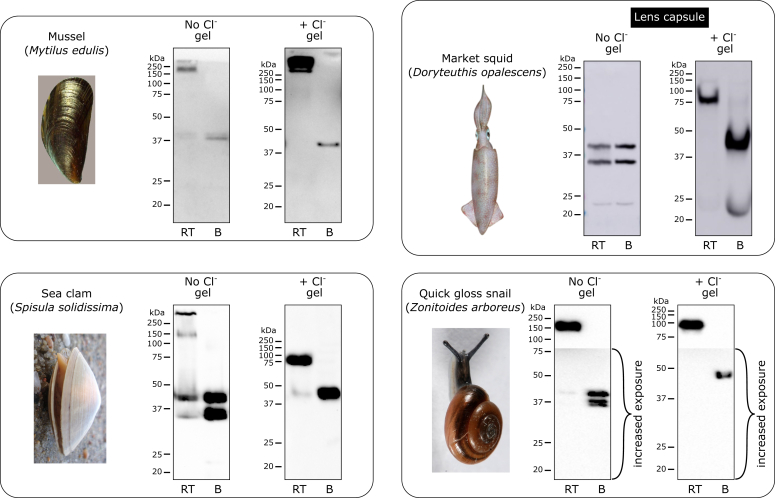
**Chloride pressure is required in some species and/or some isoforms of NC1 hexamer in Mollusca.** There is a variability of effects of chloride supplement on the stability of the NC1 hexamer ranging from complete dependence on chloride in lens capsule of the squid and almost no requirement for chloride in the snail. The mussel and clam revealed dual nature of the NC1 stability possibly pointing to two different populations of the NC1 hexamers. Animal images from Wikimedia Commons (commons.wikimedia.org) are used for illustration purposes only. B, boiled sample; RT, room temperature sample.

**Figure 6:**
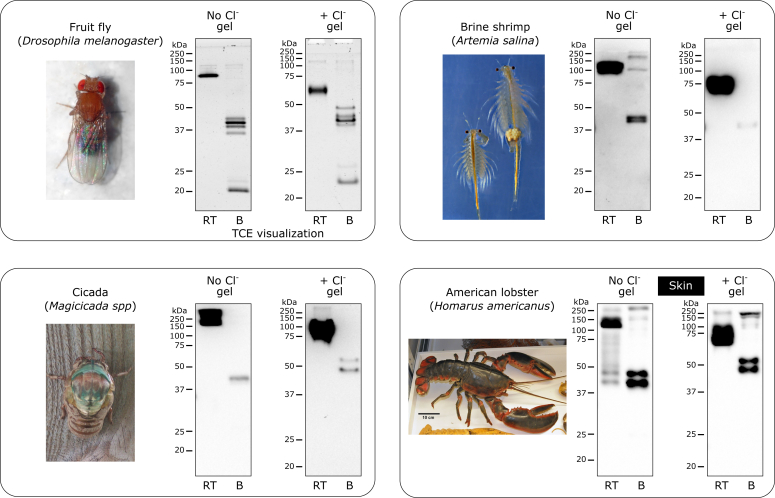
**Chloride pressure is not necessary for stability of the NC1 hexamer in Arthropoda.** All species demonstrated SDS resistance of the NC1 hexamer in the absence or the presence of chloride, thus chloride is not required for stability in Arthropoda. Fruit fly NC1 domain was imaged from the gel using the TCE in-gel visualization. Animal images from Wikimedia Commons (commons.wikimedia.org) are used for illustration purposes only. B, boiled sample; RT, room temperature sample; TCE, 2,2,2-trichloroethanol.

**Figure 7:**
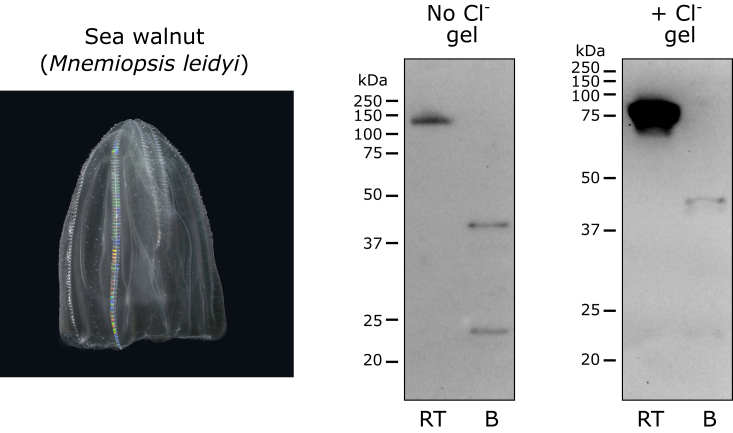
**Chloride pressure is not required for stability of the NC1 hexamer in Ctenophora.** The species demonstrated SDS resistance of the NC1 hexamer regardless of the presence of chloride. Animal images from Wikimedia Commons (commons.wikimedia.org) are used for illustration purposes only. B, boiled sample; RT, room temperature sample.

Unless otherwise noted, a small piece of tissue or small number of animals (totaling 100–200 μg) was homogenized and digested with bacterial collagenase to excise the native NC1 hexamer (Fig. 1*B*), as described in the Experimental procedures section. A universal antibody (JK2) that recognizes NC1 domain across metazoan was used for detection in Western blots. This detection method simplified the analysis in most cases without a need to purify the NC1 hexamer. Thirty-four species of animals, ranging from humans to Ctenophores, from 15 phyla were investigated (Figure 3, Figure 4, Figure 5, Figure 6, Figure 7 and S3–S13). The results show the impact of a Cl^−^ supplement to maintain the native hexamer structure that harbors Cl^−^ ions for stabilization (Fig. 1*B*).

#### Chordata phylum

Mammalian species: human, cow, mouse, and marmoset demonstrated a requirement of chloride for NC1 hexamer stability (Fig. 3). Chain-specific analysis of NC1 hexamer isolated from glomerular BM collectively revealed that all known isoforms, that is, α121/α121, α345/α345, and α121/α565, require chloride supplement for stability (Fig. 4). Other vertebrates: avian, reptiles, amphibians, and bony fishes also revealed the chloride-dependent mechanism of stabilization (Figure 3, Figure 4, Figure 5, Figure 6). In addition, iguana heart, zebrafish material, gills, eggs, and eyes of killifish (28) contained some amounts of Cl^−-^independent stable hexamers, possibly pointing to specific isoforms of collagen IV in certain tissues. Cartilaginous fish, dogfish, not only revealed the chloride-dependent mechanism but also demonstrated a significant amount of kidney-extracted hexamer to be Cl^−^ independent (Fig. S7). Finally, sea squirt, the simplest chordate, demonstrated a requirement of chloride for NC1 hexamer stability (Fig. S8).

#### Echinodermata phylum

Starfish gonads were found to be a rich source of collagen IV. The extracted NC1 hexamer demonstrated a requirement of chloride for NC1 hexamer stability (Fig. S9).

#### Mollusca phylum

Mollusca species revealed heterogeneous requirement for the presence of chloride (Fig. 5). The stability varied from an absolute dependence in the squid lens material to strongly Cl^−^-independent form in a garden snail.

#### Annelida phylum

Similar to Mollusca, two species of segmented worms demonstrated a mixed dependence on the presence of chloride in solution (Fig. S10).

#### Platyhelminthes phylum

Flatworm species revealed dual dependence on the presence of chloride in the solution (Fig. S11), whereas fresh water planaria demonstrated both isoforms, a horse crab flatworm living in the sea had mainly an isoform requiring stabilization by chloride.

#### Rotifera phylum

Rotifers were found to contain only chloride-independent isoform of the NC1 hexamer (Fig. S12).

#### Nematoda phylum

The collagen IV hexamers extracted from free-living *Caenorhabditis elegans* and parasitic *Ascaris suum* roundworms were found to be stable regardless of chloride presence in solution (Fig. S13).

#### Arthropoda phylum

Four different species of Arthropoda, that is, fruit fly, cicada, brine shrimp, and lobster, were found to contain just or predominantly chloride-independent isoform of the collagen IV hexamers (Fig. 6).

#### Ctenophora phylum

Comb jellyfish revealed chloride-independent mode of the hexamer stability (Fig. 7).

#### Cnidaria phylum

**Figure 8:**
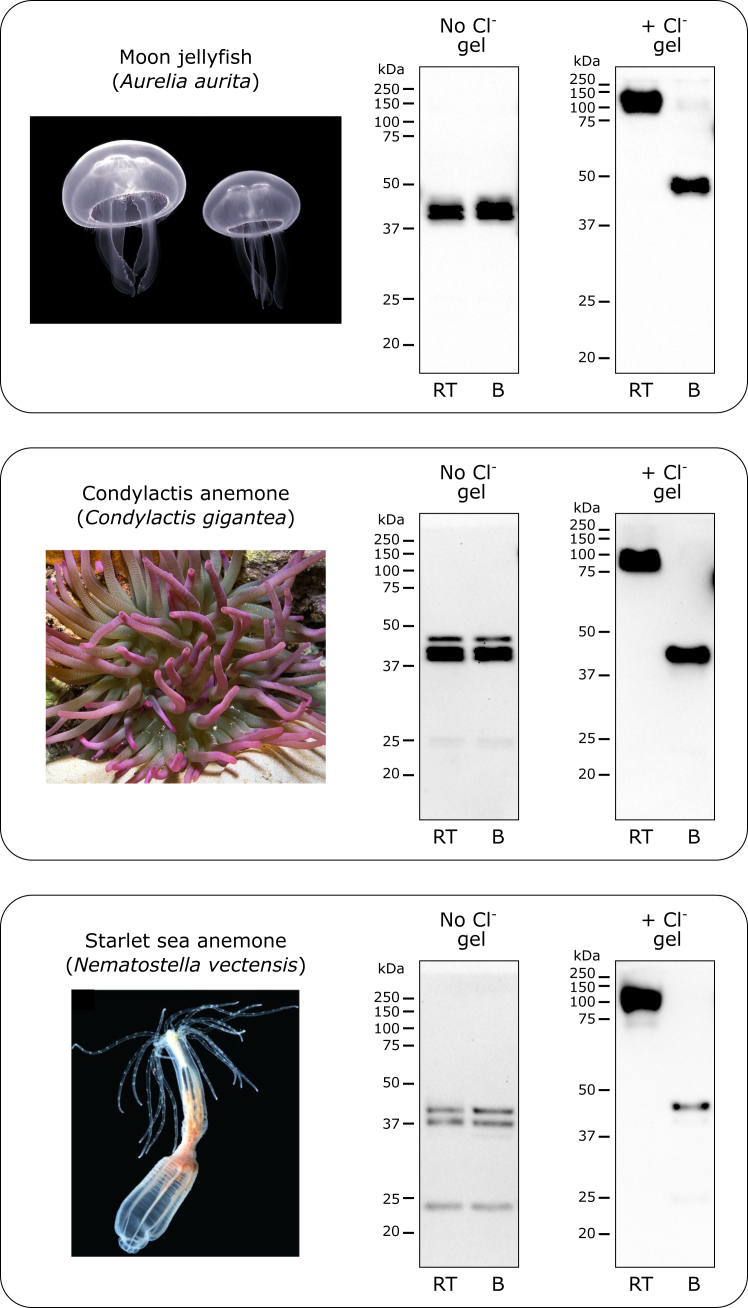
**Chloride pressure is an absolute requirement for stability of the NC1 hexamer in Cnidaria.** All three species demonstrated a requirement of chloride supplement to stabilize the NC1 hexamer. Animal images from Wikimedia Commons (commons.wikimedia.org) are used for illustration purposes only. B, boiled sample; RT, room temperature sample.

Three species of cnidarians analyzed demonstrated absolute requirement on the presence of chloride in solution for NC1 hexamer stability (Fig. 8).

**Figure 9:**
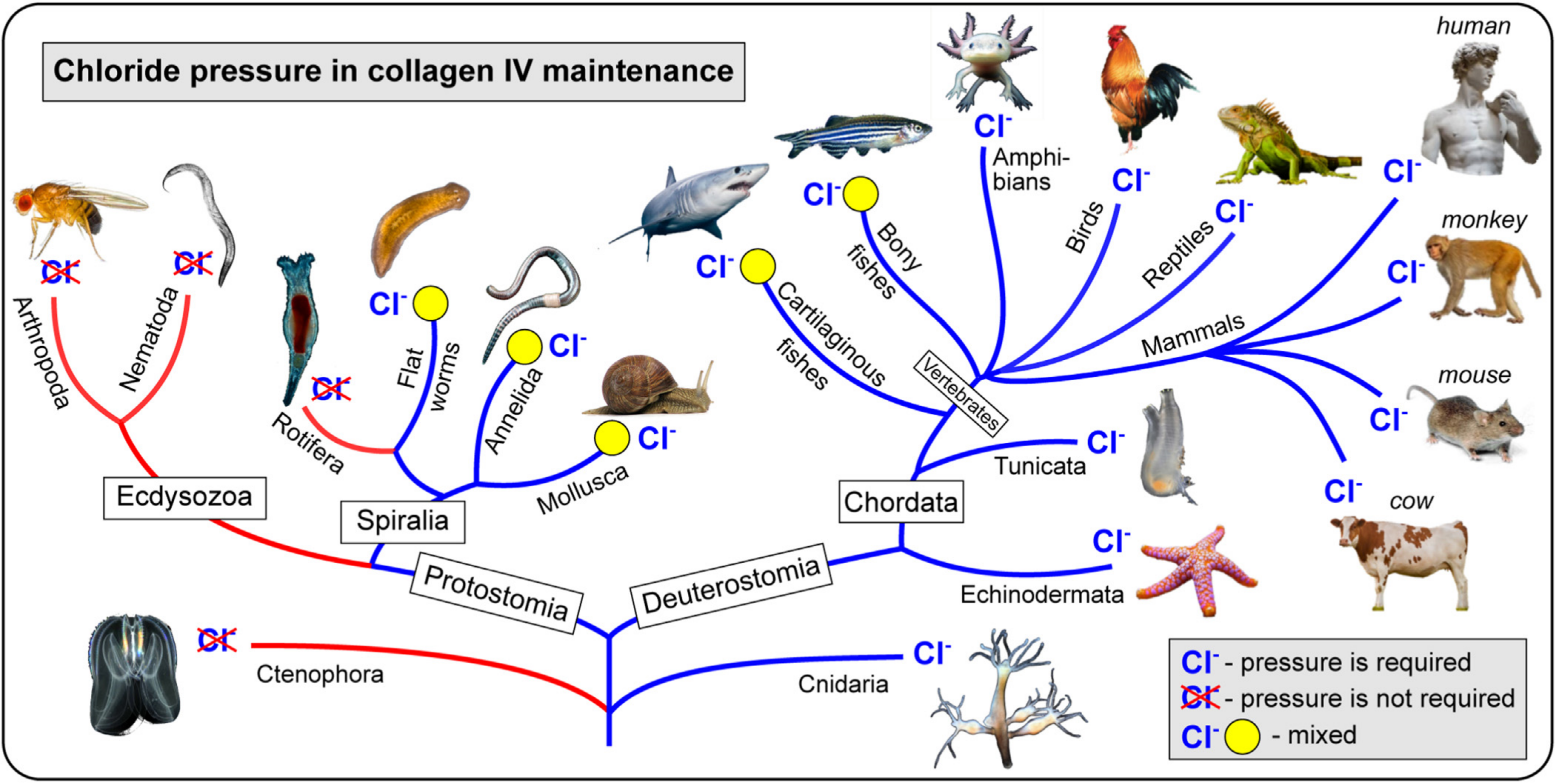
**Chloride pressure is a primordial innovation for collagen IV hexamer stability.** Discovery of the chloride pressure requirement for the hexamer stability from Cnidaria through other species to mammals demonstrated a primordial role of chloride in collagen IV evolution. No requirement of chloride pressure in Ecdysozoa and Ctenophora as well as mixed populations of the hexamers in Platyhelminthes, Annelida, Mollusca, and fishes suggests specific evolutional adaptations to environmental or tissue-specific/functional conditions. Animal images from Wikimedia Commons (commons.wikimedia.org) are used for illustration purposes only.

The role of Cl^−^ pressure throughout the animal kingdom is summarized in Figure 9.

### Cl^−^ pressure stabilizes the conformational structure of hexamer

In our recent study of collagen IV^α345^ hexamers, we found that Cl^−^ ions not only stabilize quaternary structure but also stabilize the conformation of the hexamer surface. *In vitro* studies showed that Cl^−^ ions blocked the conformational transition of hypoepitopes into neoepitopes of collagen IV^α345^ hexamers. The effect was determined by the emergence of neoepitopes that bind pathogenic anti-α3 and anti-α5 NC1 antibodies from patients with Goodpasture (GP) autoimmune disease (20). However, the juxtaposition of Cl^−^ in the context of a crosslinked hexamer posited the question of whether Cl^−^ ions alone or together with the crosslink serve as a constraint against the conformational transition.

**Figure 10:**
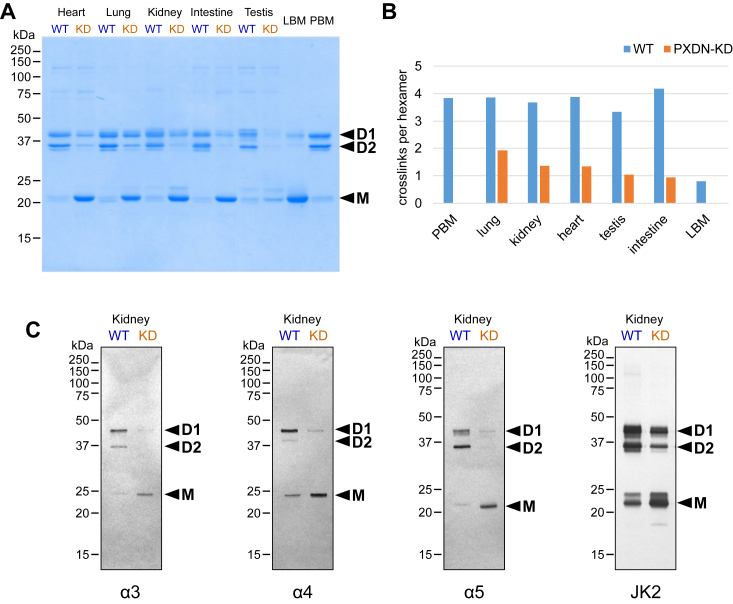
**Peroxidasin knockdown (PXDN-KD) significantly reduces crosslinking of NC1 hexamers in various organs of mouse.***A*, gel electrophoresis (regular SDS-PAGE) analysis of purified hexamers from mouse heart, lung, kidney, intestine, and testis using Coomassie staining. Monomers (noncrosslinked subunits) predominate in material from PXDN-KD mice, whereas dimers (crosslinked subunits) predominate in WT. Two controls were used, bovine lens capsule (LBM) and bovine placenta (PBM) NC1 hexamers, which predominantly noncrosslinked and crosslinked, respectively. D1 and D2 point to single and double-crosslinked dimers and M points to monomers. Nonspecific Coomassie staining reveals all isoforms of NC1 hexamers, which include α121, α345, and α121–α565. *B*, densitometric quantitation of crosslinked NC1 dimers D1 (single crosslink), D2 (double crosslink), and monomers M (no crosslinks) from the SDS-PAGE. The density values were converted into number of crosslinks per hexamer. *C*, Western blotting using α-chain-specific antibodies H31 (α3 specific), H43 (α4 specific), Mab5 (α5 specific), and a universal antibody JK2 to analyze crosslinking in the α345 hexamer. Significantly reduced crosslinking was confirmed for α3, α4, and α5 chains of NC1 hexamer isolated from PXDN-KD kidney material. LBM, lens capsule basement membrane; PBM, placenta basement membrane.

Here, we explored the Cl^−^-crosslink relationship using a peroxidasin knockdown (PXDN-KD) mouse model (29) to diminish the extent of crosslinks in the collagen IV^α345^ hexamer. The NC1 hexamers were excised from various organs of WT and PXDN-KD mice by collagenase digest, purified, and characterized by SDS-PAGE (Fig. 10). Comparison of gel patterns for PXDN-KD with WT hexamers showed a major reduction in dimers and a corresponding increase in monomers as a consequence of PXDN knockdown (Fig. 10*A*). The results reflect a substantial decrease in number of crosslinks per hexamer and a concomitant increase in uncrosslinked hexamers in all tissues (Fig. 10*B*). We probed the hexamers with chain-specific antibodies and found that crosslinks were also diminished in the α345 NC1 hexamer (Fig. 10*B*).

**Figure 11:**
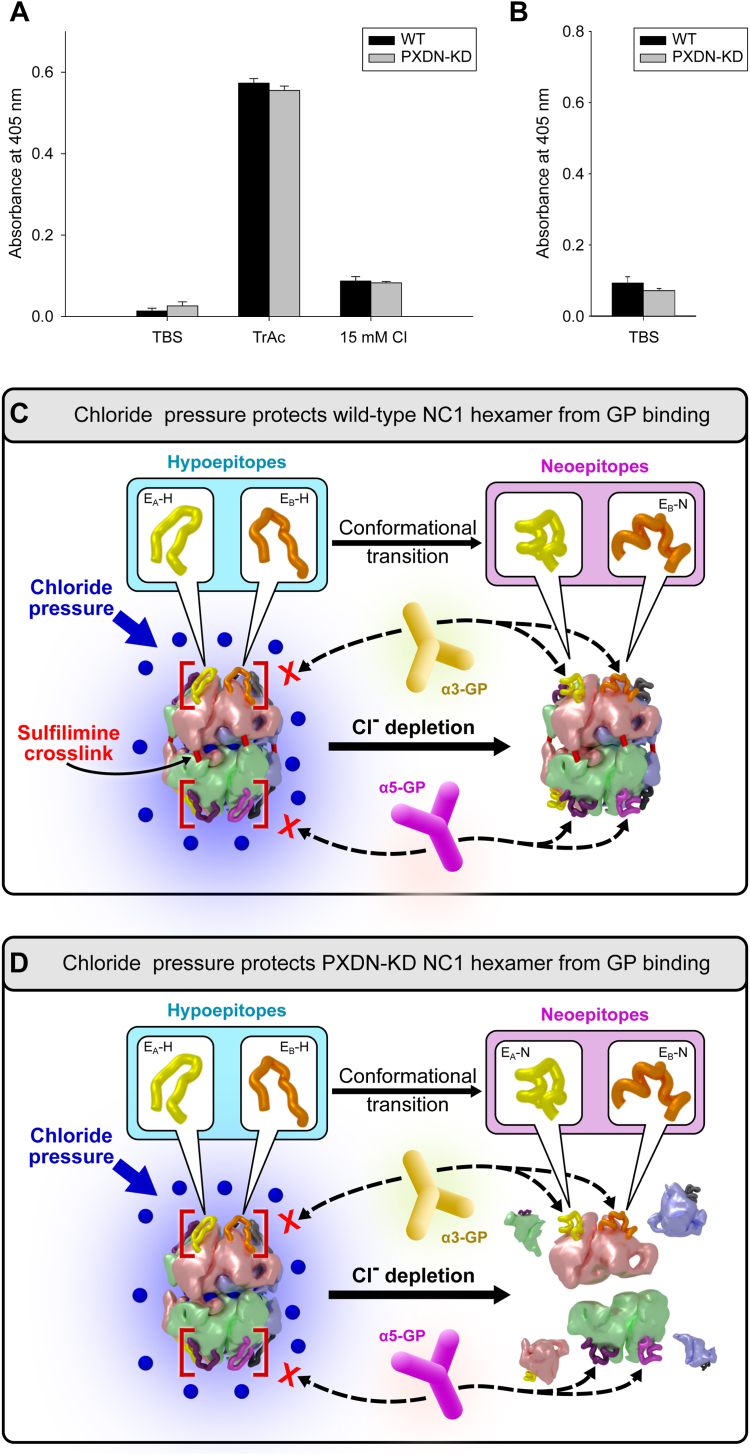
**Chloride pressure protects kidney collagen IV α345 hexamers from binding to Goodpasture (GP) autoantibodies despite significant decrease in the number of sulfilimine crosslinks.***A*, the antigen-coated plates were probed with a3-specific GP antibodies for binding under conditions with high chloride, no chloride, and little chloride concentrations. GP reactivity of PXDN-KD kidney hexamers in chloride-rich buffer (Tris-buffered saline [TBS]) was not different from WT hexamers despite greatly reduced number of crosslinks. Strong increase of reactivity was observed in Cl-free buffer (Tris-acetate), comparable for both WT and PXDN-KD mice. Even moderate chloride concentration of 15 mM significantly protected against the GP antibodies binding. *B*, second independent experiments confirmed no GP reactivity at high chloride concentration. *C* and *D*, schematic illustrations of conformational and structural fragility and GP neoepitope formation in cases of significant sulfilimine crosslinking (*C*), which prevent dissociation of the hexamer and lack of crosslinking (*D*), which is accompanied by dissociation of subunits. In both cases, GP neoepitopes are formed equally well upon loss of chloride pressure. Coloring of NC1 chains is *light red* for α3, *light blue* for α4, and *light green* for α5. PXDN-KD, peroxidasin knockdown.

To determine the effect of Cl^−^ on conformation, we measured the binding of GP antibodies to α345 hexamers from kidneys of WT and PXDN-KD mice (Fig. 11, *A* and *B*). The ELISA results showed that antibody binding, in the absence or the presence of Cl^−^, was identical for naturally crosslinked and crosslink-deficient hexamers. This result demonstrates that Cl^−^ pressure, alone and independent of sulfilimine crosslinks, is sufficient to stabilize the hexamer surface conformation (Fig. 11, *C* and *D*), revealing a role of Cl^−^ in stabilizing both quaternary structure and conformation. This finding further highlights the importance of chloride saturation on the hexamer structure and reflects the conformational plasticity of the hexamer surface (20). In GP, this conformation can be perturbed by exogenous triggers leading to production of autoantibodies. In Alport syndrome, ∼10% of missense variants occur within the NC1 domain (30) that can cause conformational changes disrupting function. Potentially, small-molecule therapeutics acting on the hexamer surface can restore function.

## Discussion

Here, we determined whether Cl^−^ ions stabilize collagen IV scaffolds across the animal kingdom. We posited this role based on previous findings that the assembly and stability of mammalian NC1 hexamers, a key assembly and structural domain of scaffolds, is Cl^−^ dependent (Fig. 1) (15, 17, 20). To this end, we developed a simple *in vitro* method of SDS-PAGE (Fig. 2) to determine the impact of solution Cl^−^ on the penultimate step of hexamer assembly (Fig. 1). SDS-PAGE analysis demonstrated that crosslinked NC1 hexamer exists in all animals, indicating that collagen IV scaffolds are expressed across animalia. Hexamers were characterized from 34 animal species across 15 major phyla, from basal cnidarians to mammals.

We found that Cl^−^, at a physiological concentration, was required for maintenance of the quaternary hexamer structure across all phyla, except for Ctenophora, Ecdysozoa, and Rotifera (Fig. 9). One such exception, a fruit fly, was the subject of thorough scrutiny in a separate study presented in an accompanying article. The results revealed an evolutional adaptation of the mechanism of assembly and stability with respect to physiologically low chloride concentration in flies. In Platyhelminthes, Annelida, Mollusca, and fishes, another exception exists wherein the stability of a second hexamer population does not require Cl^−^ (Fig. 9). These exceptions suggest specific adaptations to environmental or tissue conditions. In a study of hexamers from PXDN-KD mice, we found that Cl^−^ ions not only stabilize quaternary structure but also the conformation of the hexamer surface. Collectively, our findings revealed that the chloride concentration—“chloride pressure”—on the outside of cells is a primordial innovation that drives the assembly and dynamically stabilizes and maintains the quaternary structure and conformation of the NC1 hexamer of collagen IV scaffolds (Fig. 1).

**Figure 12:**
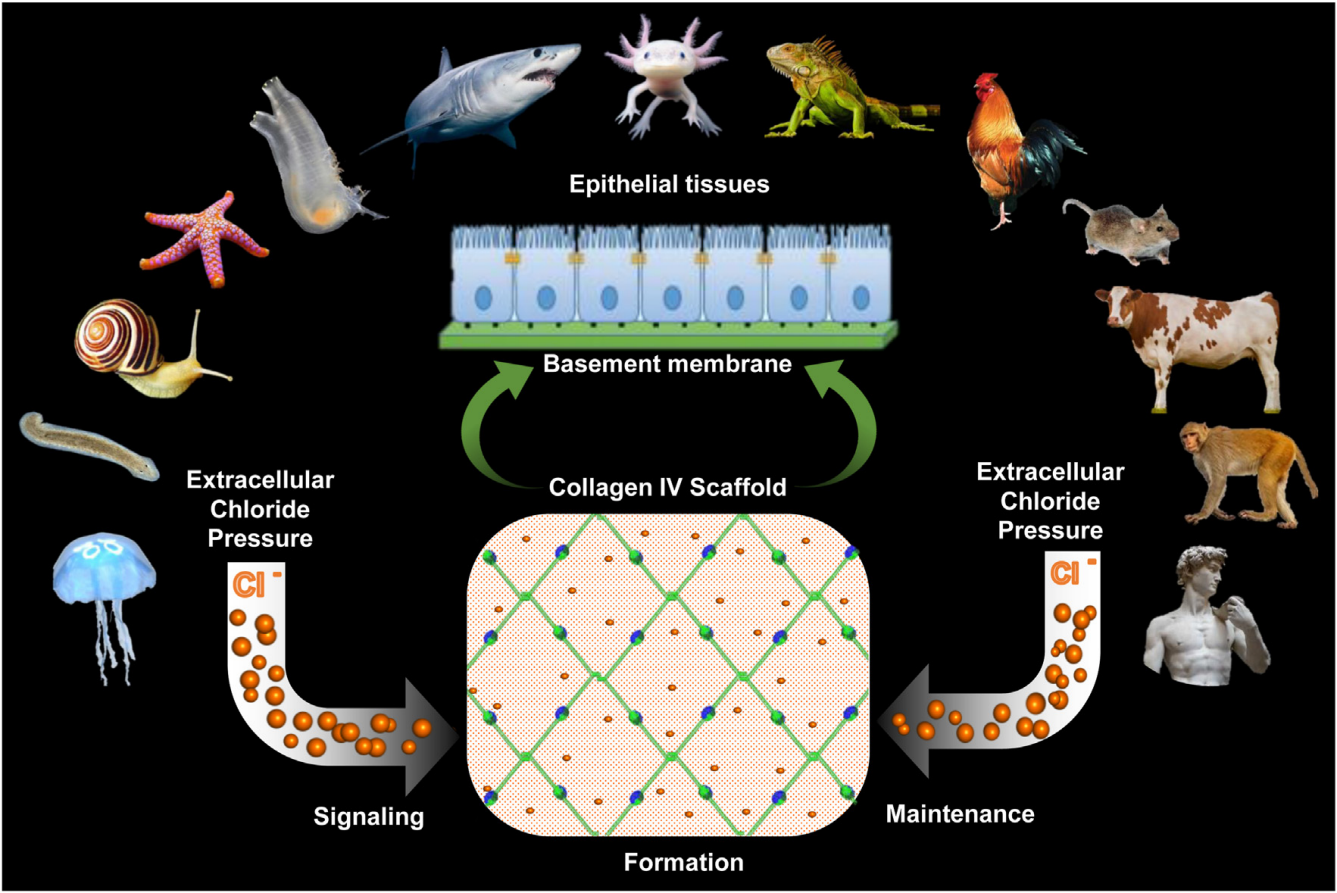
**Chloride pressure is a primordial innovation for the assembly and maintenance of collagen IV scaffolds.** This study demonstrated that maintenance of sufficient chloride concentration (pressure) is key for integrity of collagen IV scaffold beginning from Cnidaria. Together with earlier findings on the role of chloride in the assembly of collagen IV scaffold, we conclude that chloride pressure is a fundamental invention for the assembly and maintenance of collagen IV scaffolds. Animal images from Wikimedia Commons (commons.wikimedia.org) are used for illustration purposes only.

The ocean is thought to be a cradle of life. Ocean water is a rich source of chloride ions (0.54 M). Cell membrane is not only an enclosure for biological processes but also an active gradient maker for molecules including chloride ions. Chloride concentration inside the cell is low but high in the extracellular space. Unique properties of chloride ions to accept multiple hydrogen bonds enabled protein evolution and collagen IV scaffold assembly and maintenance. The location and dynamic nature of “chloride pressure” emerged as an elegant way to signal assembly and stabilize collagen IV scaffolds on the outside of cells. In turn, the scaffolds enable the assembly of a fundamental architectural unit of epithelial tissues—a BM attached to polarized cells that enabled the genesis of tissues and organs and animal evolution (Fig. 12). Yet, the molecular mechanisms that underlie normal scaffold function and dysfunction in disease remains obscure.

## Experimental procedures

### Sources of animals, tissues, and samples

#### Mammalian species

Murine (*Mus musculus*) kidneys were collected from humanely euthanized laboratory mice, WT strain C57BL/6 (approved by Vanderbilt’s Institutional Animal Care & Use Committee, protocol M1900063-00); the NC1 hexamer of human (*Homo sapiens*) kidney was from a previously purified frozen stock (20); bovine (*Bos taurus*) kidneys were purchased from a local butcher shop in Nashville; marmoset kidneys (*Callithrix jacchus*) were collected with the approval from the SA Pathology/Central Adelaide Health Network Animal Ethics Committee in 2014 under approval #68/11.

#### Avian species

Rooster (*Gallus gallus domesticus*) liver and kidneys were obtained from a farmer’s market in Arizona; chorioallantoic membrane was collected from 15-day fertilized eggs purchased from a local farm in Nashville.

#### Reptile species

Frozen food-grade meat of snapping turtle (*Chelydra serpentina*), liver of caiman (*Caiman crocodilus*), and decapitated green iguana (*Iguana iguana*) were purchased from Exotic Meat Market, Inc. (https://www.exoticmeatmarkets.com).

#### Amphibian species

Kidneys from axolotl (*Ambystoma mexicanum*) and African clawed frog (*Xenopus laevis*) were gifts from Drs Prayag Murawala and Louise A. Rollins-Smith, respectively.

#### Osteichthyes (bony fishes) species

Zebrafish (*Danio rerio*) material was a gift from Dr James G. Patton; banded killifish (*Fundulus heteroclitus*) was the field collected near Bar Harbor.

#### Chondrichthyes (cartilaginous fishes) species

Dogfish (*Squalus acanthias*) was a gift from Dr Michelle D Bailey.

#### Tunicata species

Sea squirt (*Ciona intestinalis*) was purchased from Gulf of Maine, Inc (gulfofme.com).

#### Echinodermata species

Starfish (*Asterias rubens*) was purchased from Gulf of Maine, Inc (gulfofme.com).

#### Mollusca species

Mussel (*Mytilus edulis*), sea clam (*Spisula solidissima*), and market squid (*Doryteuthis opalescens*) were purchased from a local seafood market in Nashville; quick gloss snail (*Zonitoides arboreus*) was field collected in Nolensville.

#### Annelida species

California blackworms (*Lumbriculus variegatus*) were purchased from Worm Man's Worm and Crickets Farm (https://wormman.com); leeches (*Hirudo medicinalis*) were purchased from Speedy Worm & Minnesota Muskie Farm (shop.speedyworm.com).

#### Platyhelminthes species

Culture of brown planaria (*Dugesia tigrina*) was purchased from Ward's Science (www.wardsci.com; catalog no.: 470180-236); horseshoe crab flatworm (*Bdelloura candida*) was purchased from Gulf Specimen Marine Laboratories, Inc (gulfspecimen.org; catalog no.: PL-310).

#### Rotifera species

Rotifers (*Brachionus plicatilis*) were purchased from Reed Mariculture, Inc (reefnutrition.com).

#### Nematoda species

Roundworms (*C. elegans*) were collected from laboratory stocks; the NC1 hexamer of the large roundworm of pigs (*A. suum*) was from a previously purified frozen stock (3).

#### Arthropoda species

Fruit fly (*Drosophila melanogaster*) whole animals were a gift from Andrea Page-McCaw (Vanderbilt University), and bulk material was purchased as a crude insoluble fly extract from *Drosophila* Genomics Resource Center (dgrc.bio.indiana.edu, stock #1661280); adult culture of brine shrimp (*Artemia salina*) was purchased from Northeast Brine Shrimp, LLC (www.livebrineshrimp.com); cicada (*Magicicada* spp) was field collected in Nashville; American lobster (*Homarus americanus*) was purchased from a local farm at Bar Harbor.

#### Ctenophora species

Comb jellyfish (*Mnemiopsis leidyi*) was field collected for a previous study (2). The purified sample of the NC1 hexamer of comb jelly (*M. leidyi*) was from a previous study (2).

#### Cnidaria species

Starlet sea anemone (*Nematostella vectensis*) was from a laboratory stock; condylactis anemone (*Condylactis gigantea*) was purchased from a local aquarium store; moon jellyfish (*Aurelia aurita*) was from a laboratory stock.

### Isolation of NC1 domain for Western blotting

The animals or tissues of 0.1 to 0.2 g wet weight were homogenized using metal bug beads homogenizer system in 2 ml tubes filled with 0.5 ml ice-cold Tris-buffered saline (TBS) containing 0.1% Tween-20. Homogenate was pelleted by centrifugation at 16,000*g* for 10 min at 4 °C, supernatant was discarded, and material was washed (resuspended and centrifuged) with 0.5 ml of TBS with Tween-20 four times. At the final step, insoluble material was resuspended in 0.250 ml of collagenase digestion buffer (31) supplemented with 150 mM NaCl and containing 100 μg/ml of collagenase. The suspension was incubated overnight at 37 °C while tumbling in a sealed tube. Soluble portion was collected after centrifugation at 16,000*g* for 10 min and analyzed by SDS-PAGE using either regular or NaCl-supplemented systems described in section "Chloride-supplemented SDS-PAGE".

### Isolation of NC1 domain for SEC, purification, and further analyses

This is a modified protocol (31). All buffers were supplemented with 150 mM NaCl to prevent chloride depletion from the NC1 hexamer during purification. All steps were done at 4 °C if not stated otherwise. The starting material (4 g of wet weight) was previously frozen at −80 °C and smashed using mortar and pestle while thawing. The resulting paste was resuspended in 25 ml of prechilled homogenization buffer, homogenized for 30 s using Polytron, and centrifuged at 10,000*g* for 30 min. The supernatant was discarded, the pellet was resuspended in fresh homogenization buffer using Polytron, centrifuged at 10,000*g* for 30 min, and this step was repeated two more times. A fresh buffer (20 ml) containing 10 mM Tris–HCl, pH 7.5, 1% deoxycholate (dissolved before adding NaCl!), and 150 mM was prepared at room temperature (20–25 °C), and the pellet was resuspended and incubated at room temperature for 1.5 to 2 h while mixing. The insoluble material was pelleted by centrifugation at 12,000*g* for 30 min at room temperature, and the supernatant was carefully removed. The thoroughly drained pellet was resuspended in 20 ml prechilled 25 mM Hepes, 150 mM NaCl, 25 mM 6-aminocaproic acid, 5 mM benzamidine–HCl, pH 7.5, and centrifuged at 12,000*g* at 4 °C for 30 min. The supernatant was discarded, and the previous step was repeated two more times. The final pellet was resuspended in 10 ml of the same buffer supplemented with 10 mM CaCl_2_ and 6 μg/ml collagenase and tumbled in a sealed container at 37 °C for 16 h. Finally, the suspension was centrifuged at 12,000*g* for 30 min, and the supernatant containing the NC1 domain released by collagenase digest was collected for further purification and analyses.

### Chloride-supplemented SDS-PAGE

A regular Laemmli system of Tris–glycine SDS-PAGE (32) was modified to include 150 mM NaCl in the running buffer and 150 mM NaCl in 2× SDS sample buffer (65 mM Tris–HCl, pH 6.8, 2% SDS, 25% [w/v] glycerol, and 0.01% bromophenol blue). A single gel was run at 100 V for 2 h using Mini-PROTEAN Tetra Vertical Electrophoresis Cell (Bio-Rad) filled at the maximum level with the outside buffer and intensive stirring to avoid global and local overheating of running samples.

In special cases, a Bis–Tris system was used (33), where the Mops running buffer for supplemented with 150 mM NaCl, and samples were prepared using the Laemmli 2× sample buffer supplemented with 400 mM NaCl. A single gel was run using the same cell and conditions at 80 V for 3 h.

### Western blotting

Upon completion of electrophoresis, regular and chloride-supplemented SDS gels were subjected to heating in the running buffer containing 0.1% SDS for 3 min to ensure complete denaturation of SDS-resistant species. The gels were then cooled to room temperature and subjected to a regular procedure of transfer to nitrocellulose membrane and Western blotting using NC1-specific JK2 antibody from Yoshikazu Sado, Shigei Medical Research Institute. JK2 monoclonal antibody was raised to the NC1 domain of human α3 chain but exhibited broad reactivity toward NC1 domains from different animal species (2, 3). The following chain-specific antibodies were used to detect α1 (H11), α2 (H22), α3 (Mab3), α4 (H43), α5 (H52), and α6 (B66) chains of bovine NC1 and α3 (H31), α4 (H43), and α5 (Mab5) chains of mouse NC1. Rat antihuman monoclonal antibodies, H11, H22, H33, H43, and H52, were from Chondrex (34). Mouse antibovine monoclonal antibodies mAb3 and mAb5 were from Wieslab. Rat antibovine monoclonal antibody B66 was from Yoshikazu Sado.

### Species analysis for Cl^−^ effect on SDS stability

#### Mammalian species

Straight collagenase digest of murine (*M. musculus*) kidney and partially purified NC1 hexamer from a kidney of common marmoset (*C. jacchus*) were run on the regular and “salty” gels, blotted, and detected using the JK2 antibody. Purified NC1 hexamers from kidneys of human (*H. sapiens*) and cow (*B. taurus*) were run on the regular and “salty” gels and Coomassie stained.

#### Avian species

Liver and kidney of rooster (*Gallus gallus domesticus*) as well as a 15-day-old chorioallantoic membrane were used to extract the NC1 hexamer. After collagenase digest, the NC1 hexamer was purified and probed on the regular and chloride-supplemented gels. The specific NC1 bands were detected on Western blot with the JK2 antibody.

#### Reptile species

Muscle tissue from turtle (*C. serpentina*), liver from caiman (*Caiman crocodilus*), and heart and kidney from iguana (*Iguana iguana*) were collagenase digested to release the NC1 domain from the insoluble matrix. The supernatant was analyzed on the gels, and the NC1 bands were detected by the JK2 antibody on the Western blot.

#### Amphibian species

Kidneys from axolotl (*A. mexicanum*) and African clawed frog (*X. laevis*) were homogenized and subjected to collagenase digest. The solubilized material was analyzed by Western blot with JK2 antibody.

#### Osteichthyes (bony fishes) species

Zebrafish (*D. rerio*) material was homogenized, collagenase digested, and solubilized material was partially purified by SEC before Western blot analysis; banded killifish (*F. heteroclitus*) organs were homogenized, subjected to collagenase digest, and solubilized material was analyzed by Western blot with JK2 antibody.

#### Chondrichthyes (cartilaginous fishes) species

NC1 hexamers from lens capsule, kidney, and intestine of dogfish (*S. acanthias*) were purified and analyzed on the Western blot using the JK2 primary antibody.

#### Tunicata species

Whole animal of sea squirt (*C. intestinalis*) was homogenized and subjected to collagenase digest. The soluble fraction was run on the gels, and the NC1 bands were detected by using the JK2 antibody after blotting. For chloride-supplemented SDS-PAGE instead of a regular Tris–glycine gel, we used Bis–Tris gel, which allowed better separation of the hexamer band from the dimer.

#### Echinodermata species

Starfish (*A. rubens*) gonads were found to be a rich source of the NC1 hexamer. Collagenase digest of homogenized gonads was directly analyzed on the gels, and the NC1 bands were recognized by the JK2 antibody after blotting.

#### Mollusca species

Collagenase digests of mussel (*M. edulis*) and sea clam (*S. solidissima*) whole tissues were run on the regular and “salty” gels, blotted, and detected using the JK2 antibody. Partially purified NC1 hexamers from lens capsules of market squid (*D. opalescens*) and from whole animals of the quick gloss snail (*Z. arboreus*) were run on the regular and “salty” gels, blotted, and detected using the JK2 antibody.

#### Annelida species

Collagenase digests of homogenates of segmented worms, California blackworms (*L. variegatus*), and leeches (*H. medicinalis*) were run on the gels and detected on Western blots by using the JK2 antibody.

#### Platyhelminthes species

Whole animal homogenates of brown planaria (*D. tigrina*) and horseshoe crab flatworm (*B. candida*) were collagenase digested and partially purified over the SEC. The hexamer peaks were concentrated and analyzed on the gels. Brown planaria NC1 domain was recognized by the JK2 antibody after blotting, whereas horseshoe crab flatworm NC1 domain was visualized in the gel using the 2,2,2-trichloroethanol (TCE) approach (35).

#### Rotifera species

Whole animal homogenate of rotifera (*B. plicatilis*) was subjected to collagenase digest and partial purification on the size-exclusion column. The hexamer fraction was run on the gels, and bands of NC1 domain were detected on Western blot using the JK2 antibody.

#### Nematoda species

NC1 hexamer from roundworm *C. elegans* was partially purified after the collagenase digest using the SEC. The protein bands on the gel were visualized using the TCE in-gel visualization (35). The NC1 hexamer purified from large roundworm of pigs (*A. suum*) (3) was analyzed on the Western blots using the JK2 antibody.

#### Arthropoda species

Purified NC1 hexamer of fruit fly (*D. melanogaster*) was run on regular and “salty” gels and detected using the TCE in-gel visualization (35). Collagenase digests of cicada (*M*. spp), brine shrimp (*A. salina*), and American lobster (*H. americanus*) were run on the regular and “salty” gels, blotted, and detected using the JK2 antibody.

#### Ctenophora species

Partially purified NC1 hexamer of comb jelly (*M. leidyi*) was run on regular and “salty” gels, blotted, and detected using the JK2 antibody.

#### Cnidaria species

Partially purified NC1 hexamers of moon jellyfish (*A. aurita*), condylactis anemone (*Condylactis gigantea*), and starlet sea anemone (*N. vectensis*) were run on regular and “salty” gels, blotted, and detected using the JK2 antibody.

### PXDN-KD mouse model

We used the PXDN-KD mouse model with about 7% residual PXDN activity and about one-third normal sulfilimine crosslinks (29) to collect tissues for analysis. Heart, lung, kidney, intestine, and testis tissues were used for isolation of the NC1 hexamer following the modified protocol (31), where NaCl at 150 mM was added to all solutions to prevent dissociation of the noncrosslinked isoform.

### Quantification of protein bands from the SDS-PAGE

Coomassie-stained gels were imaged with the Gel Doc XR+ system (Bio-Rad), and density profiles of individual lanes were measured using the ImageJ (National Institutes of Health and the Laboratory for Optical and Computational Instrumentation, University of Wisconsin), version 1.53a software (36). Density profiles were further processed with the Fityk (Marcin Wojdyr), version 1.3.1 program (37) for baseline subtraction and integration of protein bands as a readout of quantity.

### Chloride effect on the presentation of α3 NC1 GP epitopes

Binding of affinity-purified α3-specific GP autoantibody (0.2 μg/ml) was tested using ELISA (38). Polystyrene microtiter plates (Nunc MaxiSorp; Thermo Fisher Scientific) were coated overnight with murine kidney NC1 hexamers in TBS, pH 7.4 (TBS), 20 mM Tris-acetate, 100 mM sodium acetate, pH 7.4 (TrAc), or TrAc supplemented with 15 mM NaCl and blocked with 1% bovine serum albumin. Alkaline phosphatase–conjugated goat antihuman immunoglobulin G (1:2000 dilution) was used as a secondary antibody. p-Nitrophenyl phosphate (Sigma) was used as a substrate, and the color development was monitored at 405 nm using SpectraMax-190 microplate reader (Molecular Devices).

### Data processing, statistical analysis, and figure preparation

Data processing and figures were done using the Excel (Microsoft, https://office.microsoft.com/excel), Gimp (The GIMP Development Team, https://www.gimp.org), and Inkscape (The Inkscape Project, http://www.inkscape.org/) software.

## Data availability

All data are contained within the article or supporting information.

## Supporting information

This article contains supporting information (17).

## Conflict of interest

The authors declare that they have no conflicts of interest with the contents of this article.
