# Supplementary material for: Collagen IV of basement membranes: III. Chloride pressure is a primordial innovation that drives and maintains the assembly of scaffolds
Source: J Biol Chem. 2023 Oct 4;299(11):105318. doi: 10.1016/j.jbc.2023.105318 (PMC10656227; doi:10.1016/j.jbc.2023.105318)
Supplement: Supporting Figures S1–S13 [file mmc1.docx]

**Supporting Information**

This appendix has been provided by the authors to give readers additional information about their work.

Supplement to: **Collagen IV of basement membranes: III. Chloride pressure is a primordial innovation for the assembly and maintenance of the scaffolds.**

***Authors***

Sergei P. Boudko, Aspirnauts^*^, Vadim K. Pedchenko, Elena N. Pokidysheva, Alena M. Budko, Rachel Baugh, Patrick Toby Coates, Aaron L. Fidler, Heather M. Hudson, Sergey V. Ivanov, Carl Luer, Tetyana Pedchenko, Robert L. Preston, Mohamed Rafi, Roberto Vanacore, Gautam Bhave, Julie K. Hudson, Billy G. Hudson.

*Aspirnauts: Octavia Ailsworth, ZaKylah Bryant, Camryn Cole, Jacob Edwards, Di’Andra Edwards, Sydney Farrar, Julianna Gallup, Michael Gallup, Martina Gergis, Aalia Holt, Madeline Lach, Elizabeth Leaf, Finn Mahoney, Max McFarlin, Monica Moran, Galeesa Murphy, Charlotte Myers, Connie Ni, Neve Redhair, Rocio Rosa, Olivia Servidio, Jaeden Sockbeson, Lauren Taylor.

Aspirnaut is a K-20 Science, Technology, Engineering and Math (STEM) pipeline for increasing the diversity and wellness of the STEM workforce. The holistic training approach features **guided discovery science** that is augmented with **guided professional skills development, guided self-discovery,** and **wellness training**. High school students from rural America and diverse backgrounds engage in hands-on discovery science for six weeks while in residence at Vanderbilt University Medical Center, and diverse undergraduate students engage for 10 weeks. The experience provides students with the tools and empowerment to effect positive change in themselves, their families, and communities for generations to come. **Details of the STEM pipeline are presented in Supplement No. 2.**

***Content***

Figure S1. Chloride groups 1 and 2 S-2

Figure S2. Conservation of chloride coordinating residues S-3

Figure S3*. Aves S-4

Figure S4*. Reptiles S-5

Figure S5*. Amphibians S-6

Figure S6*. Osteichthyes (bony fishes) S-7

Figure S7*. Chondrichthyes (cartilaginous fishes) S-8

Figure S8*. Tunicata S-9

Figure S9*. Echinodermata S-10

Figure S10*. Annelida (segmented worms) S-11

Figure S11*. Platyhelminthes (flatworms) S-12

Figure S12*. Rotifera (wheel animals) S-13

Figure S13*. Nematoda (roundworms) S-14

* - animal images from Wikimedia Commons (commons.wikimedia.org), Gulf Specimen Marine Laboratories, Inc.(gulfspecimen.org), and EOL (eol.org) are used for illustration purposes only.


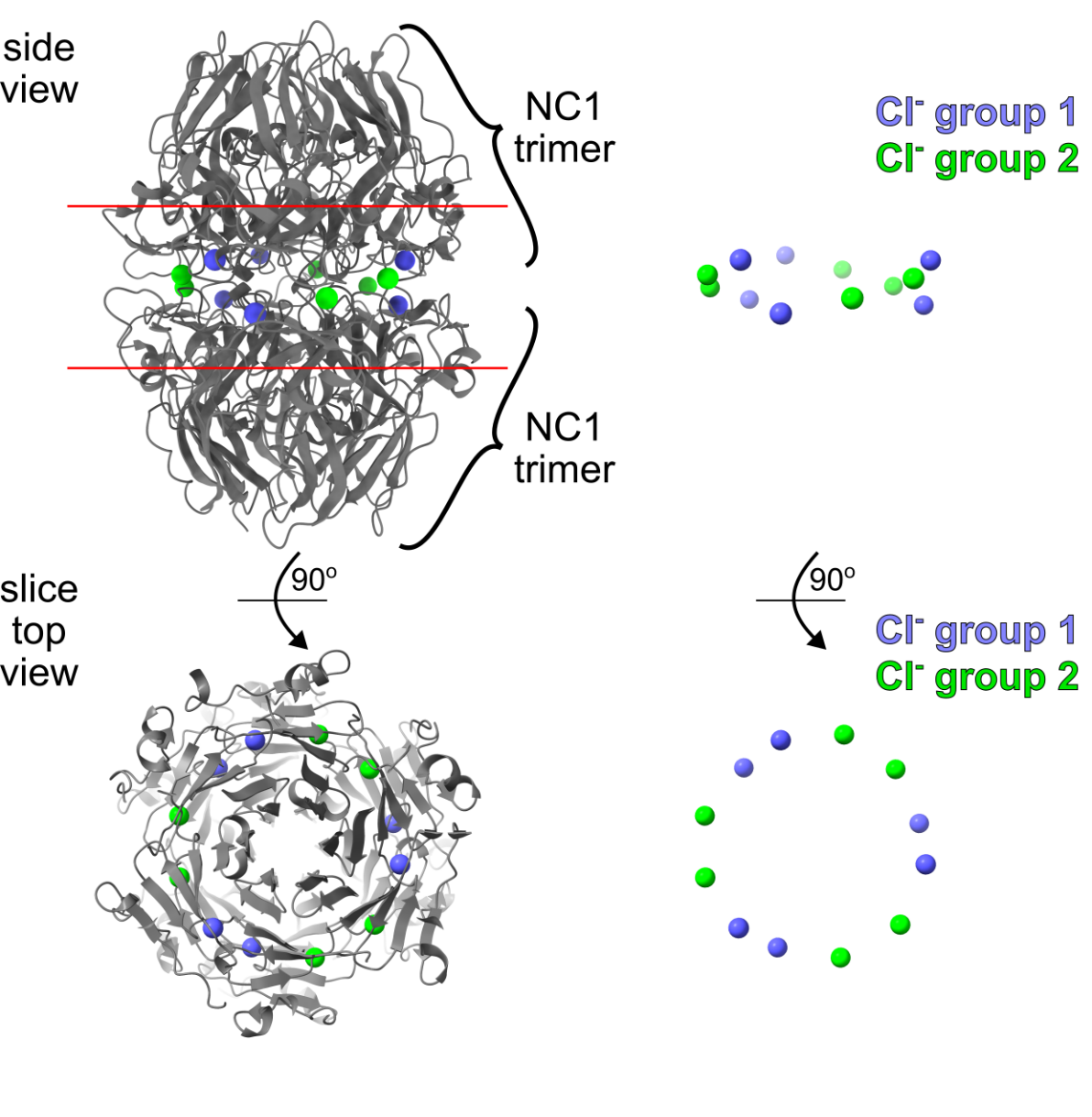


Figure S1. **Chloride groups 1 and 2**. The hexamer structure composed of two NC1 trimers. The hexamer is depicted as ribbons. Chloride ions are shown as spheres. Interaction of two NC1 trimers is coordinated by two groups of chloride ions (group 1 and group 2). Adapted from Pedchenko *et al.* (17).


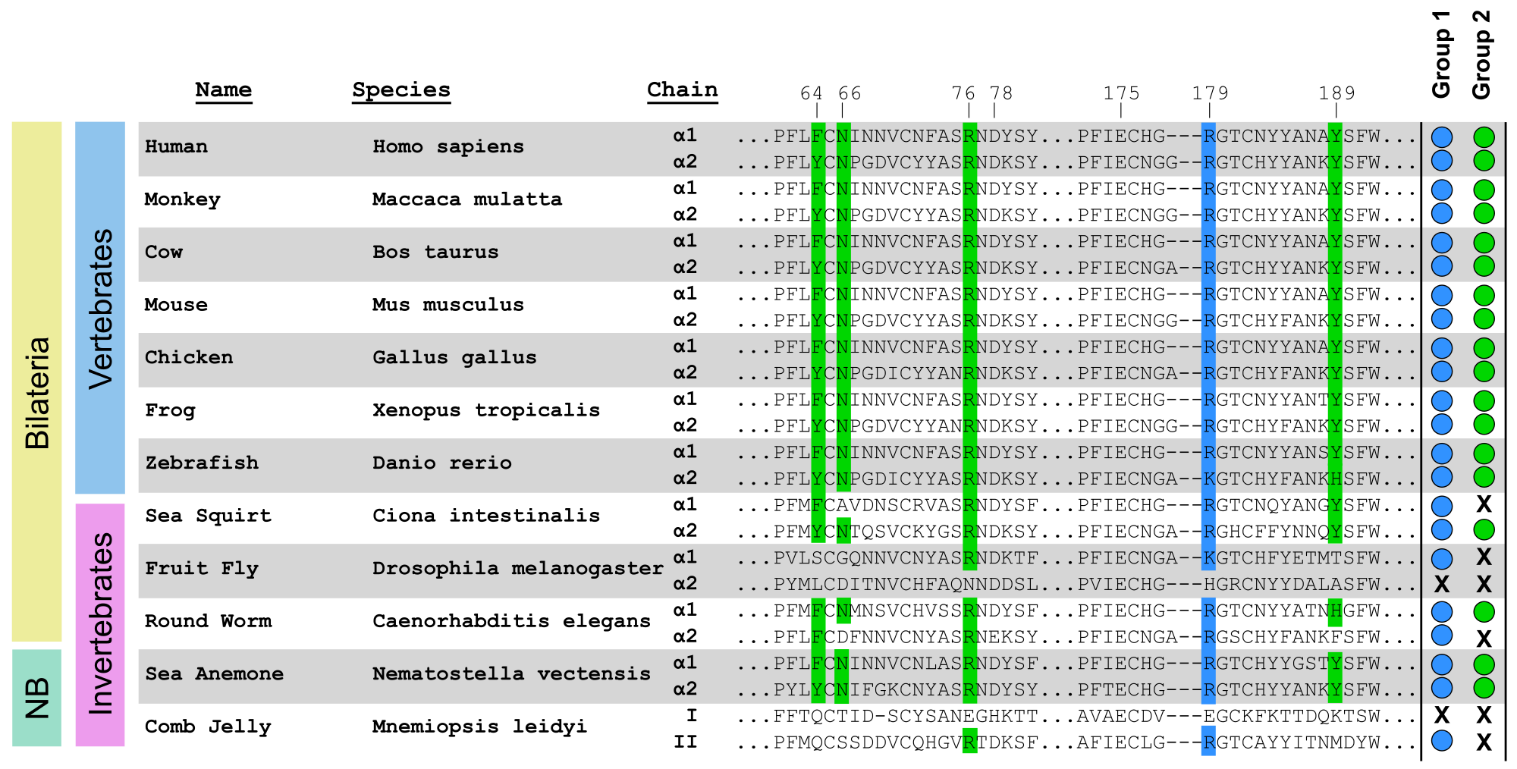


Figure S2. **Conservation of chloride coordinating residues**. Evolutionary analysis of amino acid residues of collagen IV NC1 domains that are directly involved in chloride coordination. The side chain of Arg/Lys-179 (blue) is coordinated by a group 1 chloride ion of the opposite trimer. Group 2 chloride ions coordinate side chains of residues Phe/Tyr-64, Asn-66, Arg-76, and Tyr/His-189 of both trimers (green). NB – non-bilaterian animals. Adapted from Pedchenko *et al.* (17).


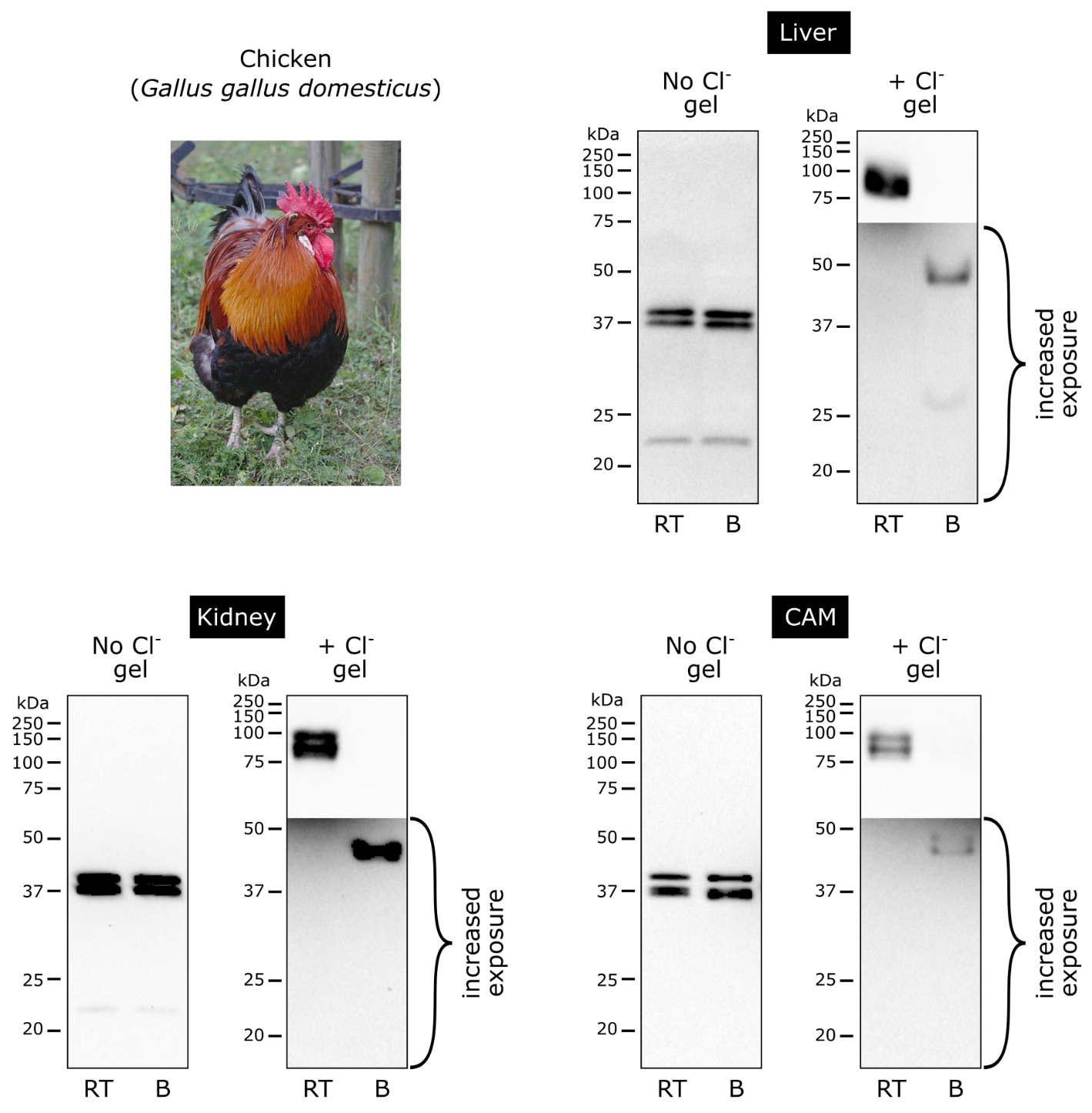


Figure S3. **Chloride pressure is required for stability of the collagen IV hexamer in chicken**. All samples which include material purified from chicken liver, kidney, and chorioallantoic membrane (CAM), revealed that the chicken NC1 hexamer is SDS-resistant only in the presence of chloride. RT – room temperature sample. B – boiled sample.


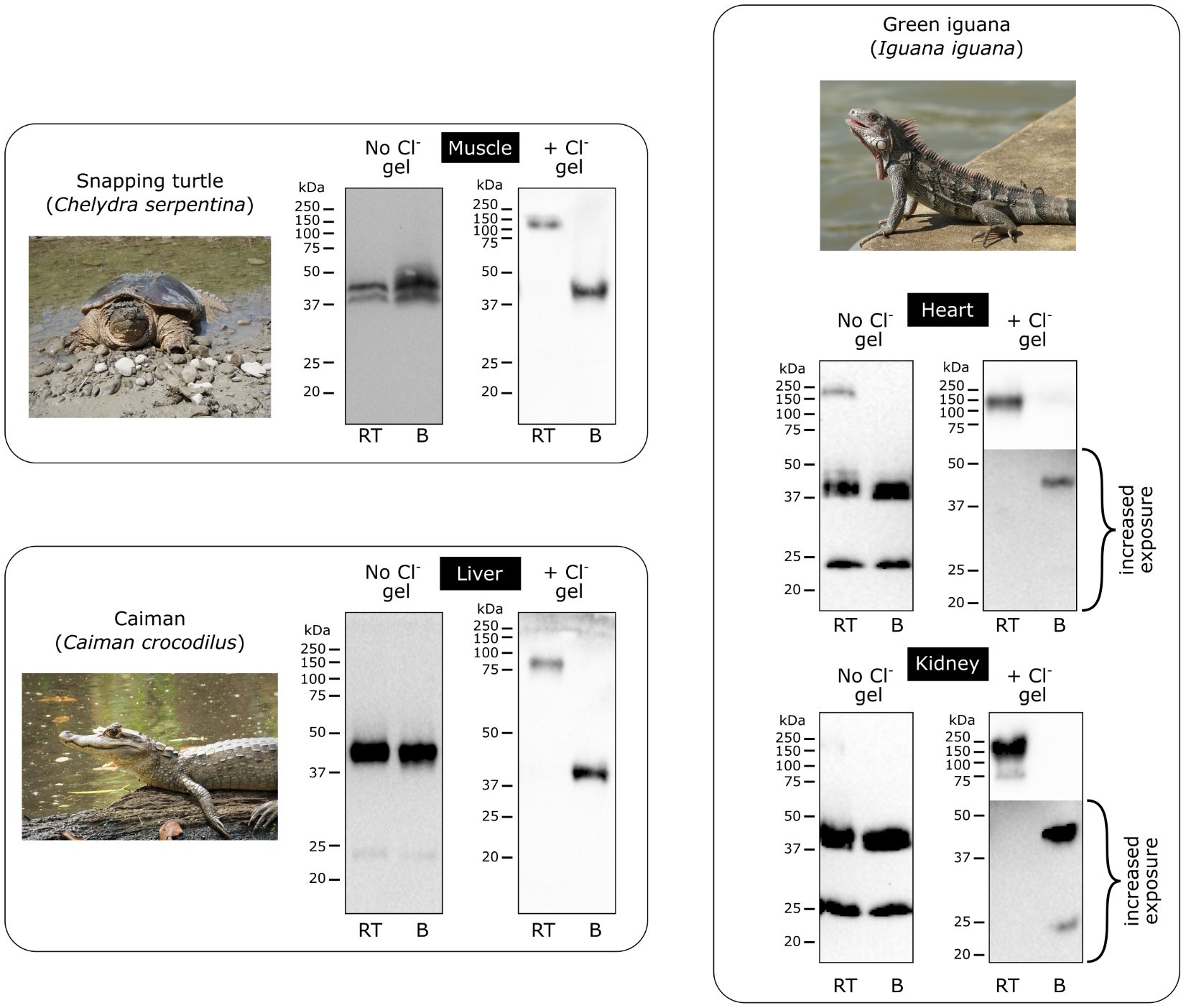


Figure S4. **Chloride pressure is required for stability of the collagen IV hexamer in Reptiles**. Majority of the NC1 hexamers require supplementation of chloride to withstand denaturing effect of SDS. A small fraction of the hexamer isolated from the heart of the iguana was SDS-resistant in the absence of chloride. RT – room temperature sample. B – boiled sample.


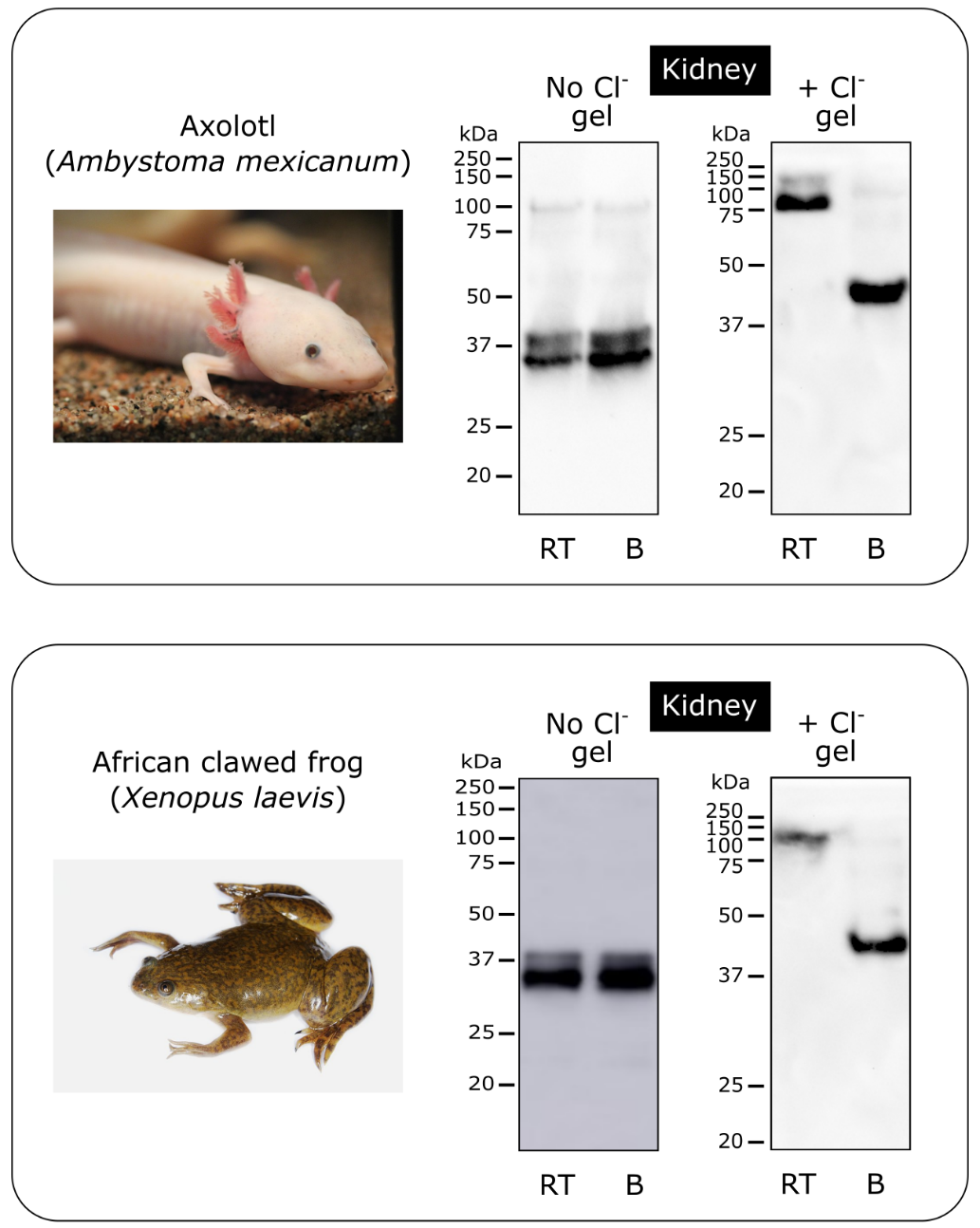


Figure S5. **Chloride pressure is required for stability of the collagen IV hexamer in Amphibians**. Both species revealed that SDS-resistance of the NC1 hexamer requires the presence of chloride. RT – room temperature sample. B – boiled sample.


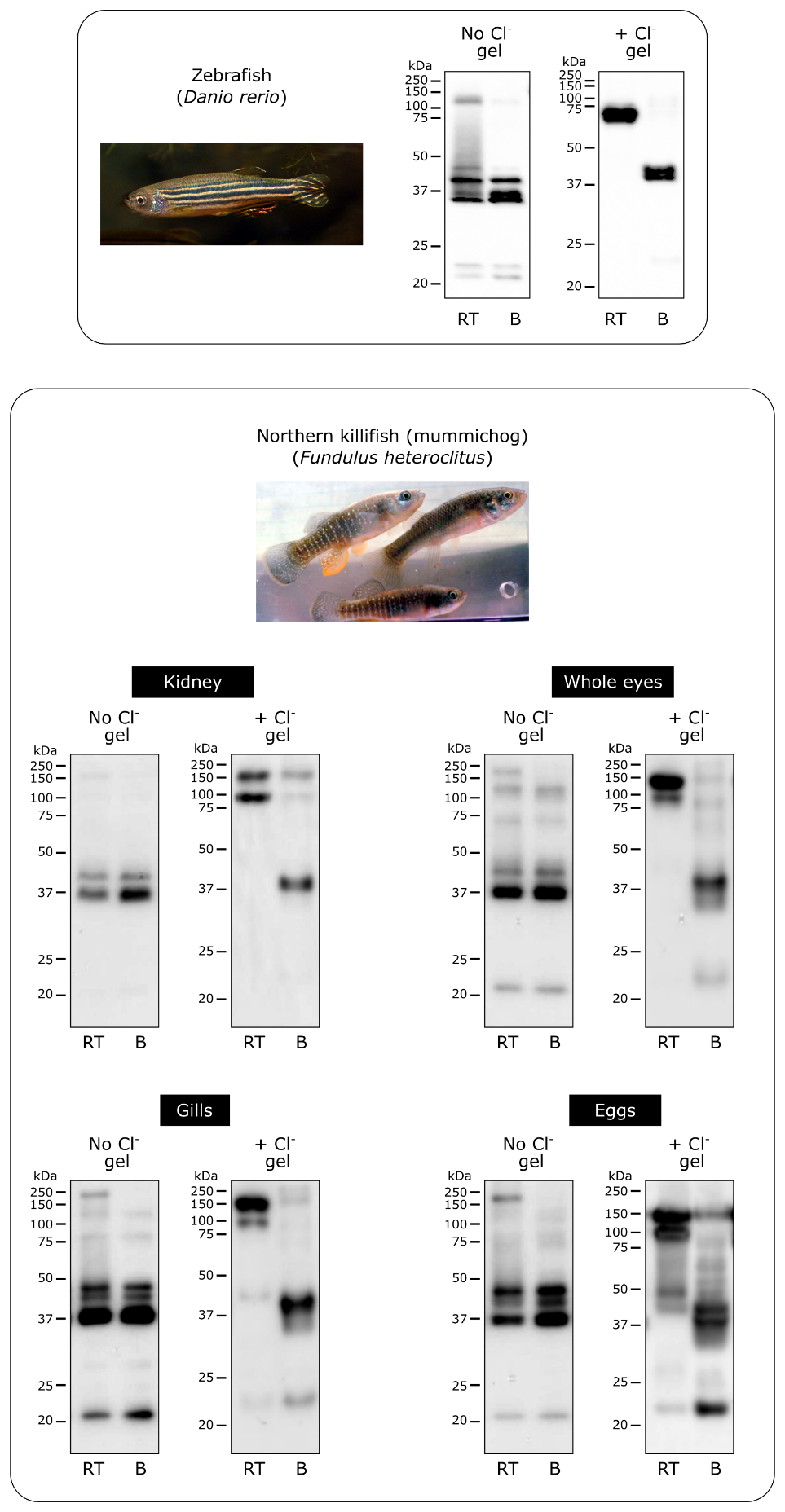


Figure S6. **Chloride pressure is required for stability of major isoforms of the collagen IV hexamer in Osteichthyes (bony fishes)**. A whole zebrafish and specific parts of a killifish were homogenized and digested with collagenase to release the NC1 domain. Significant amount of the NC1 hexamer demonstrated chloride-dependent resistance to SDS on the western blots. Nevertheless, detectable amounts of the NC1 hexamer were found to withstand denaturing power of SDS in the absence of chloride. RT – room temperature sample. B – boiled sample.


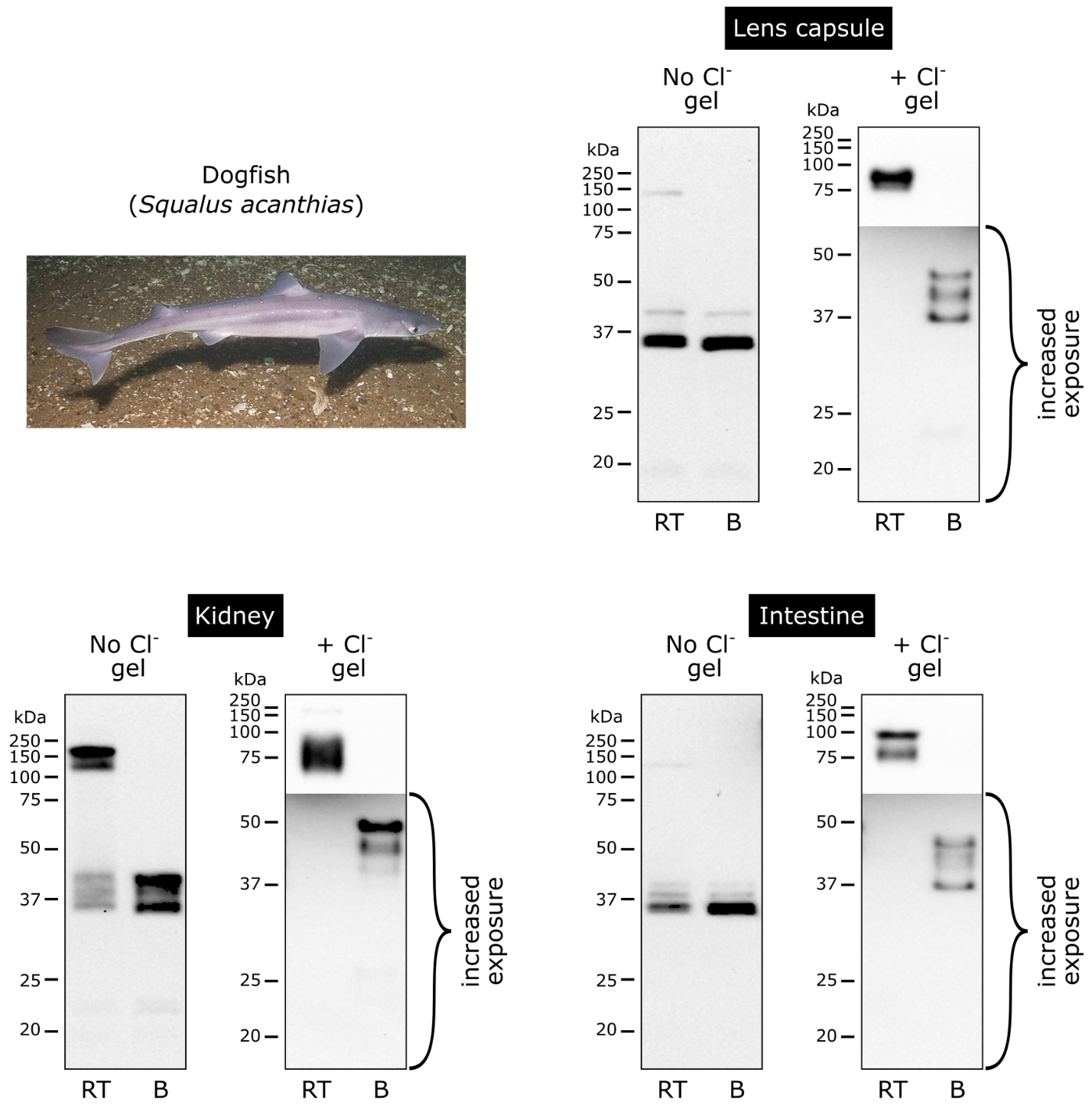


Figure S7. **Chloride pressure is required for stability of certain isoforms of the collagen IV hexamer in Chondrichthyes (cartilaginous fishes)**. Lens capsule and intestine contained predominantly the chloride-dependent isoform of the NC1 hexamer. Kidney revealed a significant amount of chloride-independent SDS-resistance. RT – room temperature sample. B – boiled sample.


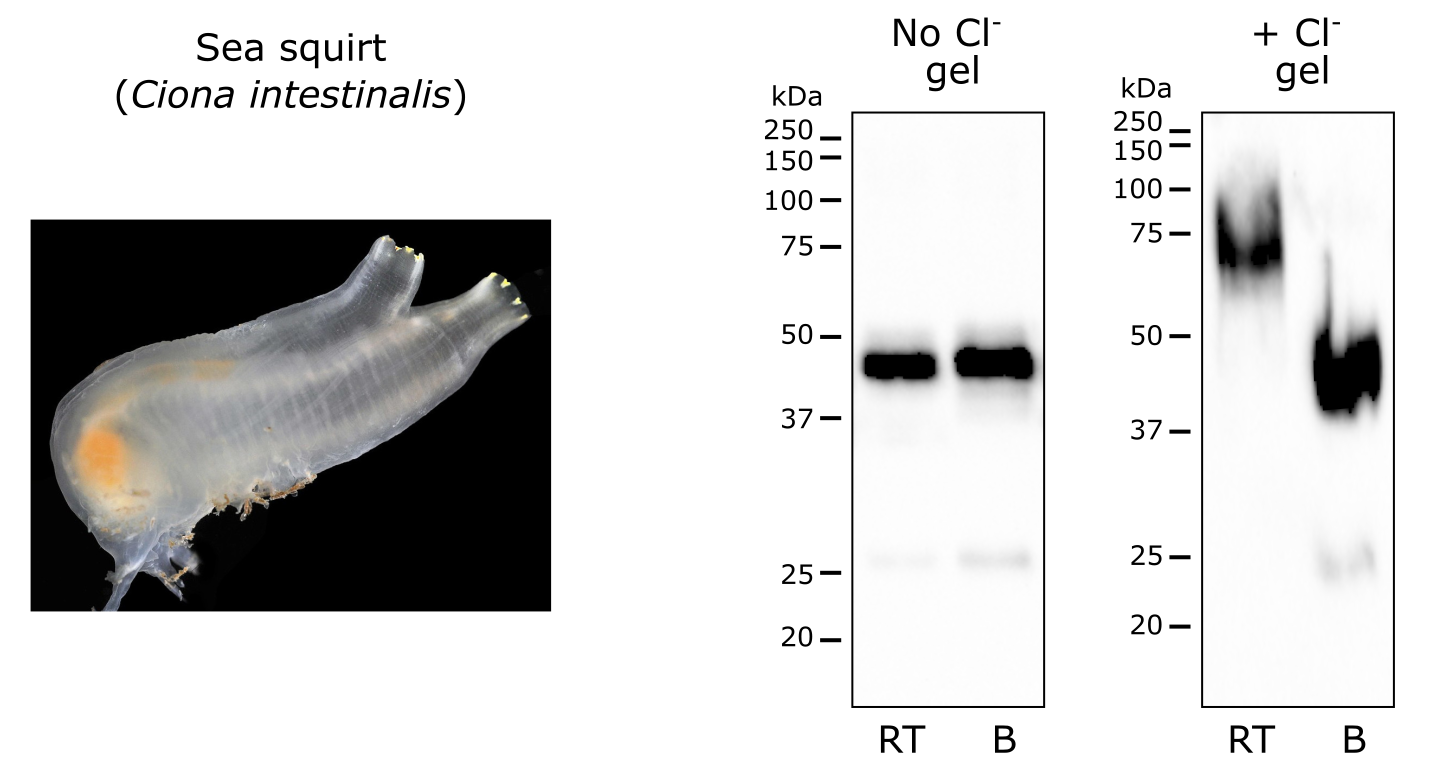


Figure S8. **Chloride pressure is required for stability of the collagen IV hexamer in Tunicata**. A primitive chordate *Ciona intestinalis* revealed chloride-dependent stability of the NC1 hexamer. For chloride-supplemented SDS-PAGE instead of a regular Tris-Glycine gel we used BisTris gel, which allowed better separation of the hexamer band from the dimer. RT – room temperature sample. B – boiled sample.


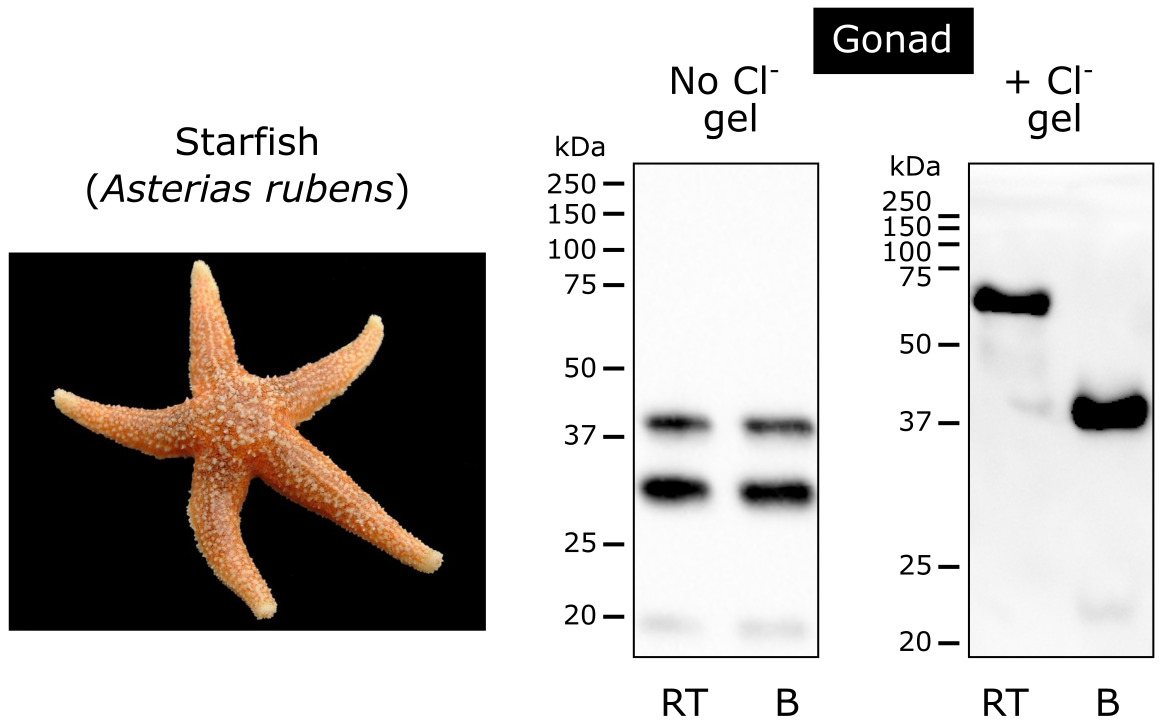


Figure S9. **Chloride pressure is required for stability of the collagen IV hexamer in Echinodermata**. Starfish gonads were found to be a rich source of collagen IV. We found that chloride pressure is required for stability of the hexamer. RT – room temperature sample. B – boiled sample.


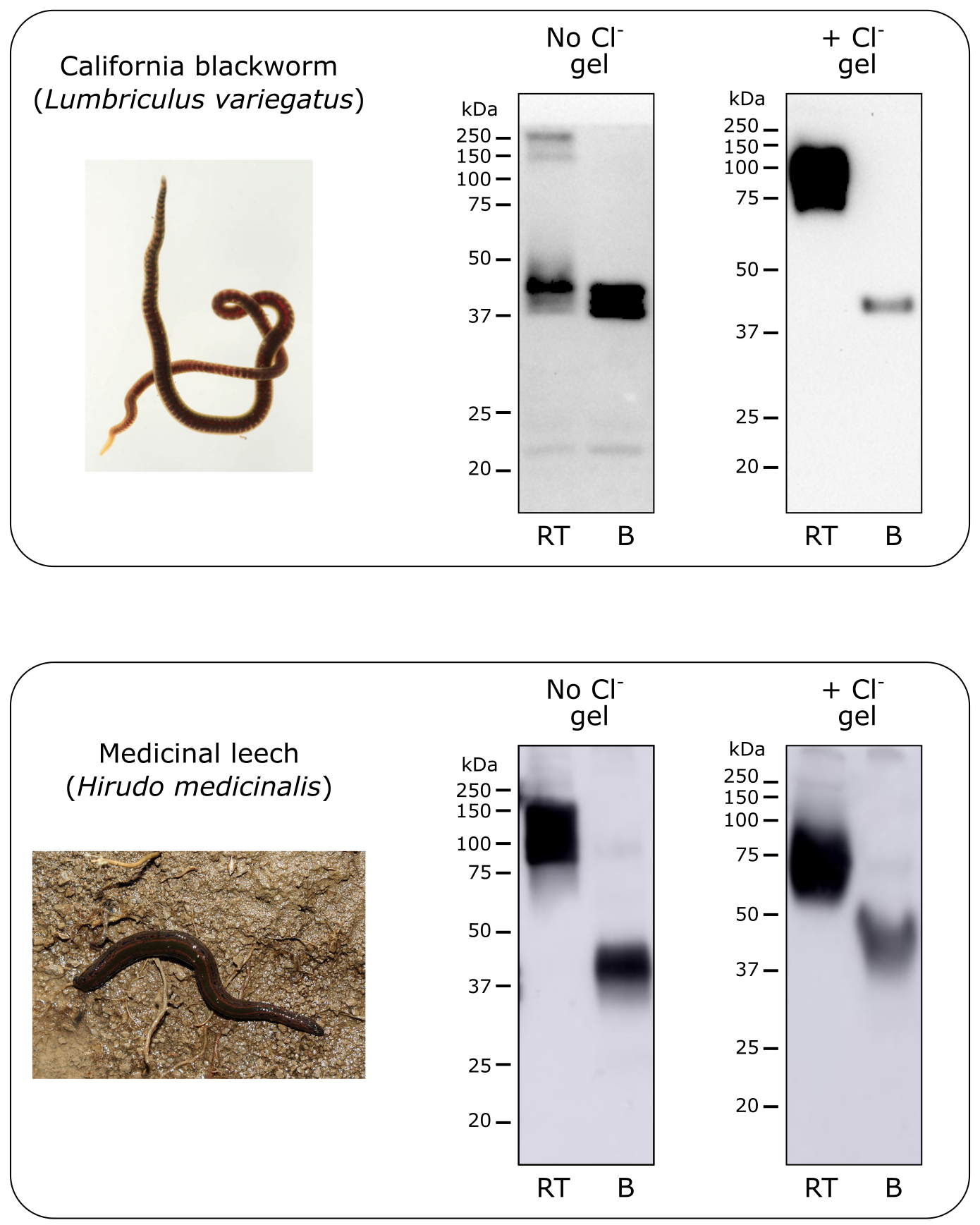


Figure S10. **Chloride pressure is required for stability of the collagen IV hexamer in certain species of Annelida (segmented worms)**. Whereas the NC1 hexamer from California blackworm (*Lumbriculus variegatus*) requires presence of chloride in solution for SDS-resistance, medicinal leech (*Hirudo medicinalis*) does not need chloride. RT – room temperature sample. B – boiled sample.


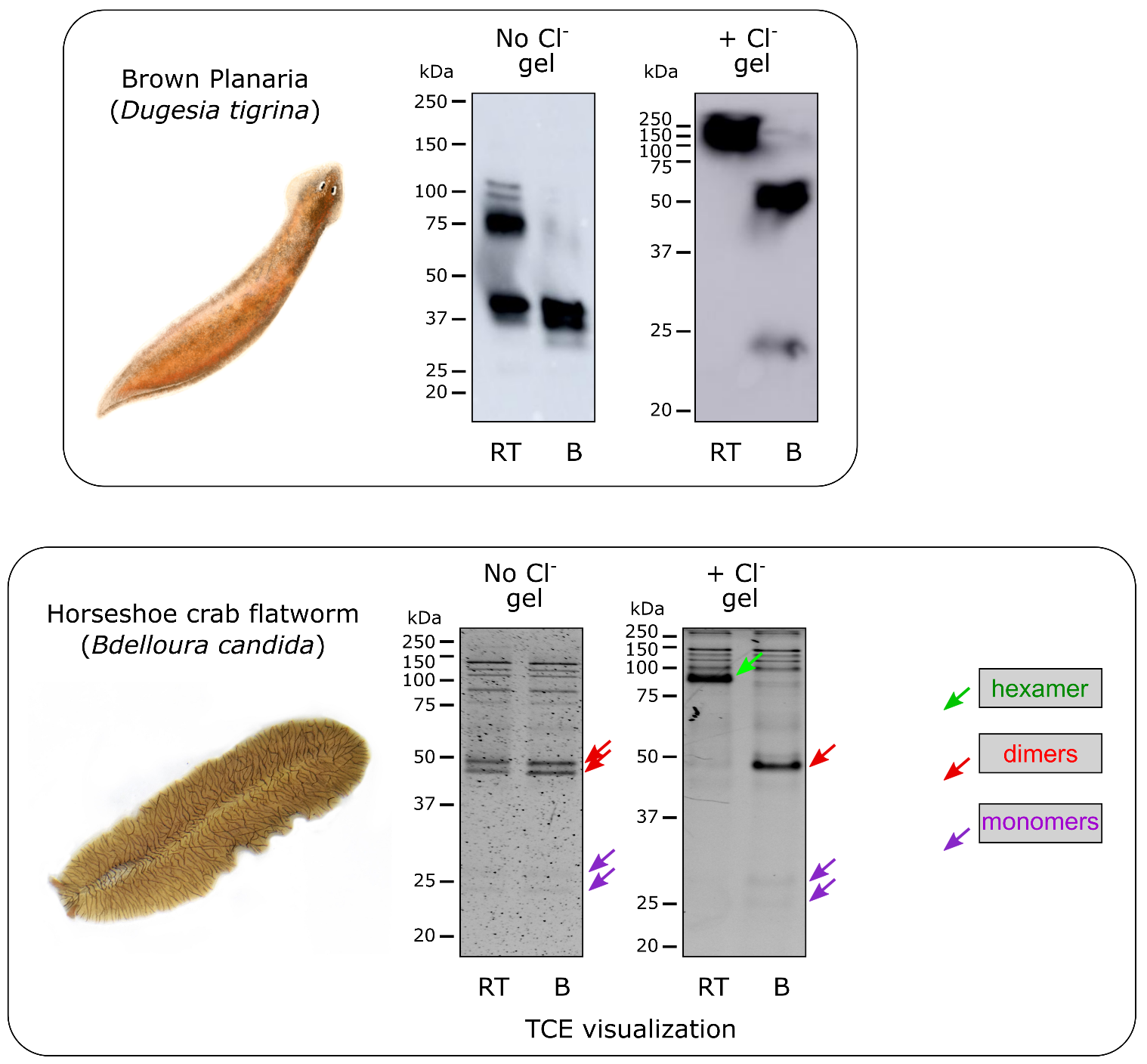


Figure S11. **Chloride pressure is required for stability of certain isoforms of collagen IV and in certain species of Platyhelminthes (flatworms)**. While the NC1 hexamer isolated from brown planaria (*Dugesia tigrina*) revealed two isoforms with and without chloride stabilization effect, horseshoe crab flatworm (*Bdelloura candida*) does not require chloride pressure. Brown planaria NC1 domain was recognized by the JK2 antibody after blotting, while horseshoe crab flatworm NC1 domain was imaged from the gel using the TCE in-gel visualization. RT – room temperature sample. B – boiled sample.


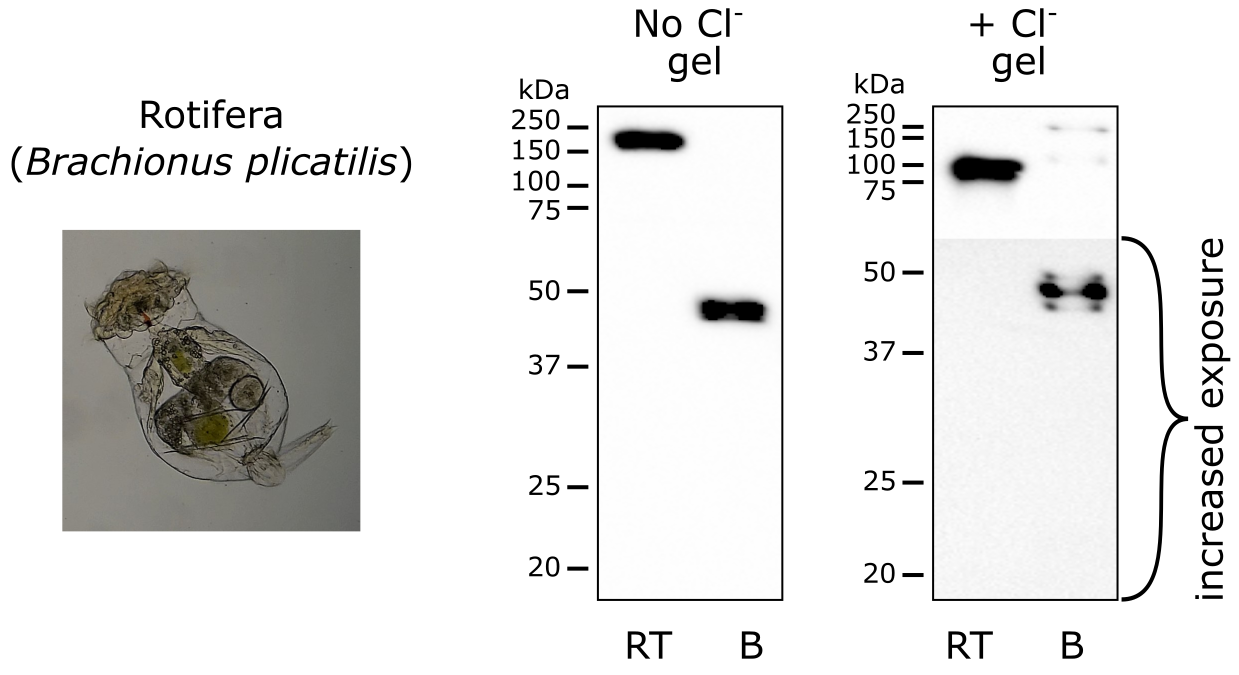


Figure S12. **Chloride pressure is not required for stability of the NC1 hexamer in Rotifera (wheel animals)**. The NC1 hexamer is SDS-resistant with or without supplementation of chloride. RT – room temperature sample. B – boiled sample.


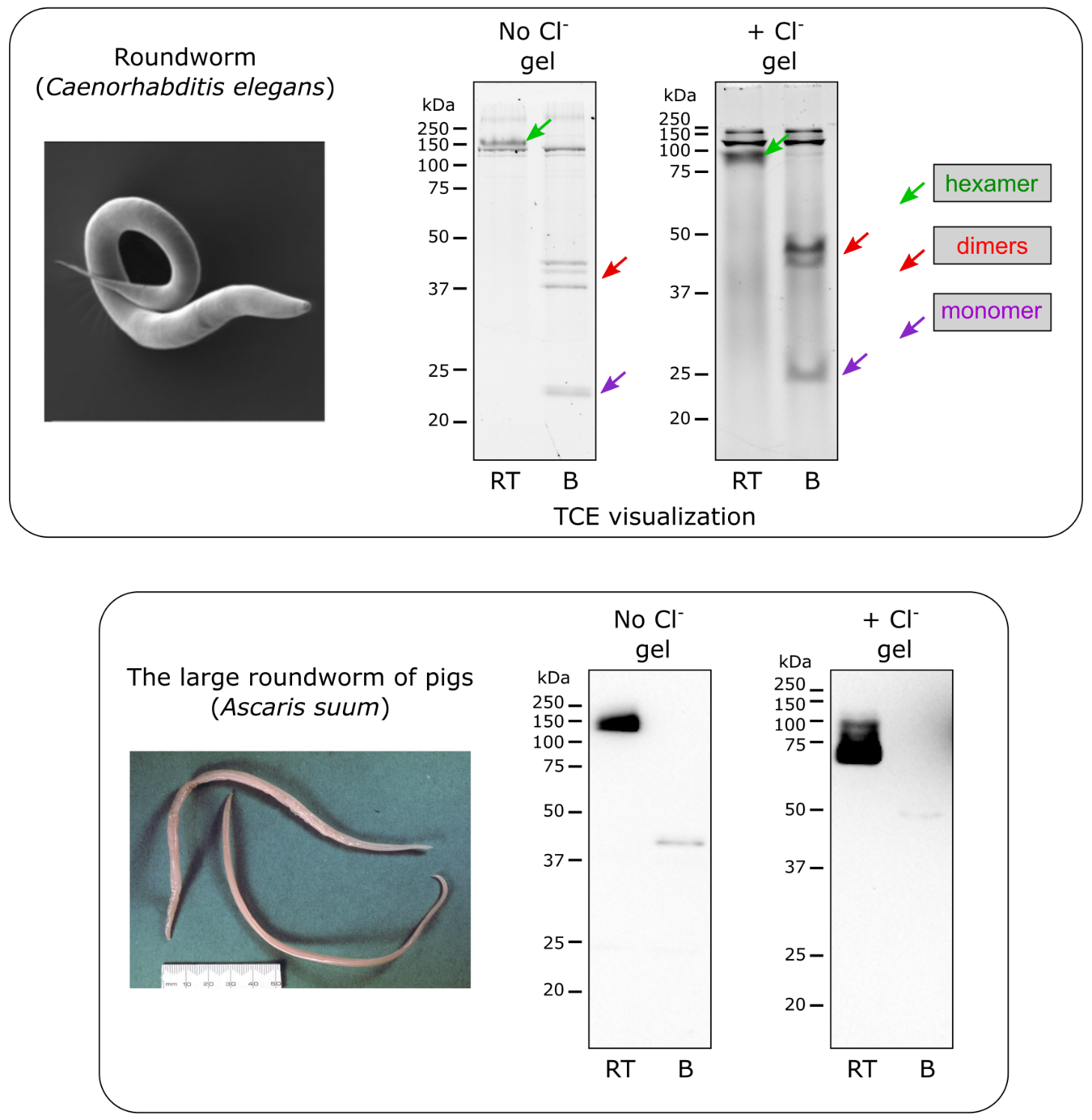


Figure S13. **Chloride pressure is not required for stability of the NC1 hexamer in Nematoda (roundworms)**. The NC1 hexamers were SDS-resistant under both chloride-free and chloride-supplemented conditions. *C. elegans* protein bands on the gel were visualized using the TCE in-gel visualization, while that of *A. suum* were detected on the western blot. RT – room temperature sample. B – boiled sample.
