# Supplementary material for: Collagen IV of basement membranes: III. Chloride pressure is a primordial innovation that drives and maintains the assembly of scaffolds
Source: J Biol Chem. 2023 Oct 4;299(11):105318. doi: 10.1016/j.jbc.2023.105318 (PMC10656227; doi:10.1016/j.jbc.2023.105318)
Supplement: Supporting Information [file mmc2.docx]

**ASPIRNAUT K-20 STEM PIPELINE FOR DIVERSITY AND WELLNESS**

**Rachel Baugh, Billy G. Hudson, Julie K. Hudson**

1. **Aspirnaut Co-Authors: An Overview**

There is a critical need to increase the recruitment and training of a diverse workforce to meet economic and industrial demand in science, technology, engineering, and mathematics (STEM) careers. STEM careers are the fastest-growing occupations in the United States, with 75% of jobs requiring some education past high school^1-3^. Individuals from diverse backgrounds face barriers that impede their entry and persistence in undergraduate STEM studies. These limitations include a lack of exposure to STEM education, qualified instruction in K-12, STEM educational resources, professional mentoring, and adverse childhood experiences.

Over the past fifteen years, we have developed the **Aspirnaut K-20 STEM pipeline for diversity and wellness** to assist high school students from rural and diverse backgrounds in overcoming these barriers. High school students engage in hands-on discovery science for six weeks while in residence at Vanderbilt University Medical Center, and diverse undergraduate students engage for ten weeks. The experience introduces the students to the world of advanced biology and chemistry, medicine, and the excitement of discovery. Training uses a holistic approach that features **guided discovery science** that is augmented with **guided professional skills development, guided self-discovery,** and **wellness training**.

In the present study, eighteen high school and five undergraduate students from ten states entered the pipeline as members of a research team. The goal was to test the hypothesis that: **chloride ions play a fundamental role in the assembly of collagen IV scaffolds, enabling tissue genesis and evolution.** Collagen IV hexamers, key assembly domains, were characterized from thirty animal species from fifteen phyla across the animal kingdom, including the basal Cnidarian. Collectively, their findings revealed a primordial function of chloride pressure that drives and maintains the quaternary and conformational structure of hexamers of collagen IV scaffolds.

1. **List of Aspirnaut Co-Authors**

| Student Name | Year on Project | Status While on Project | Hometown | University Attended |
| --- | --- | --- | --- | --- |
| Ailsworth, Octavia | 2021 | College Junior | Memphis, TN | Lincoln University |
| Bryant, ZaKylah | 2021 | College Sophomore | Lexington, KY | Kentucky State University |
| Cole, Camryn* | 2021 | HS Senior | West Helena, AR | Xavier University of Louisiana |
| Edwards, Di'Andra* | 2021 | HS Senior | Helena, AR | University of Arkansas, Conway |
| Edwards, Jacob | 2019 | College Senior | Jackson, MO | Vanderbilt University, University of Colorado Medical School |
| Farrar, Sydney | 2016, 2017, 2018 | HS Sophomore, Junior, Senior | Calais, ME | Iowa Wesleyan University |
| Gallup, Julianna* | 2022, 2023 | HS Junior, HS Senior | Sevierville, TN | Still in high school |
| Gallup, Michael* | 2022, 2023 | HS Junior, HS Senior | Sevierville, TN | Still in high school |
| Gergis, Martina* | 2021, 2022 | HS Senior, College Freshman | Nashville, TN | Davidson College |
| Holt, Aalia* | 2022, 2023 | HS Senior, College Freshman | Benton, AR | Yale University |
| Lach, Madeline* | 2021 | HS Senior | Chicago, IL | University of Pennsylvania |
| Leaf, Elizabeth* | 2022 | HS Senior | Deer Isle, ME | Mount Holyoke College |
| Mahoney, Finn | 2022 | College Junior | Tamworth, NH | Berea College |
| McFarlin, Max | 2010 | HS Senior | Malvern, AR | University of Central Arkansas |
| Moran, Monica | 2012-2017 | HS Junior, College Senior | Goodlettsville, TN | Berea College, University of Alabama Birmingham (Ph.D.) |
| Murph, Galeesa | 2010-2011 | HS Junior, HS Senior | West Helena, AR | Vanderbilt University |
| Myers, Charlotte* | 2021 | HS Junior | Temecula, CA | Massachusetts Institute of Technology |
| Ni, Connie* | 2021 | HS Senior | Chattanooga, TN | University of Pennsylvania |
| Redhair, Neve* | 2019, 2021, 2022 | HS Junior, College Freshman, College Sophomore | Tuba City, AZ | Stanford University |
| Rosa, Rocio* | 2021, 2022 | HS Junior, HS Senior | Mebane, NC | Vanderbilt University |
| Servidio, Olivia* | 2022 | HS Senior | Lewiston, ME | Lewiston High School |
| Sockbeson, Jaeden* | 2021, 2022 | HS Senior, College Freshman | Indian Island, ME | Columbia University |
| Taylor, Lauren | 2022 | Master’s Student | Grapevine, AR | University of Arkansas at Monticello |

*** Indicates Lu-Springer Research Scholars**

1. **Demographics of Aspirnaut Research Interns**

One hundred percent (100%, 23/23) of Aspirnaut™ research interns identified as individuals from diverse backgrounds. Forty-three percent (43%, 10/23) were from racial and ethnic groups underrepresented in STEM, seventy percent (70%, 16/23) from rural backgrounds, sixty-five percent (65%, 15/23) from economically disadvantaged backgrounds, and forty-eight percent (48%, 11/23) from a family with no parent/guardian having a bachelor's degree. In addition, around 25% of Aspirnaut interns over the last fifteen years have a self-reported Adverse Childhood Experience (ACE) score of four or higher, ultimately predisposing them to lifelong increased risk of cardiovascular disease, cancer, diabetes, asthma, COPD, substance abuse, mental illness, and autoimmune disease^4-5^.


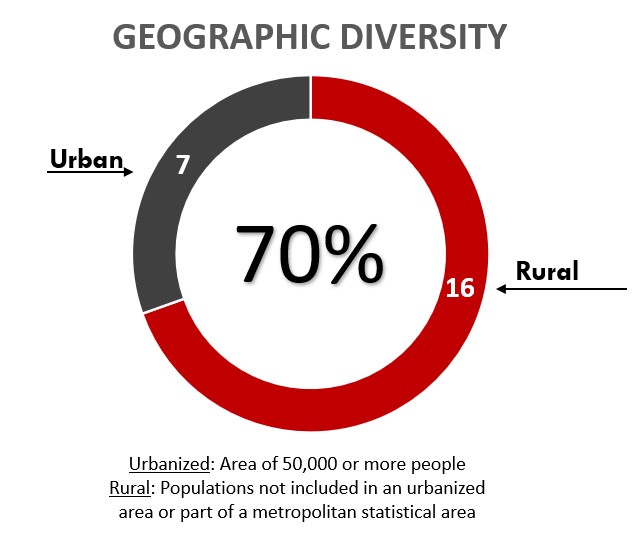

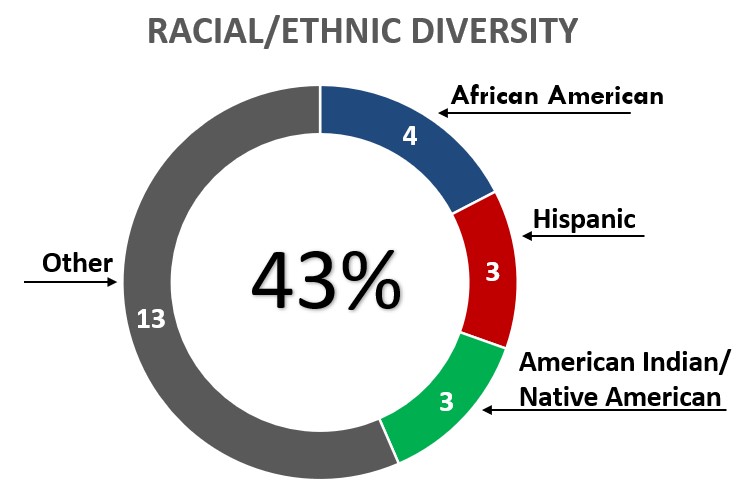


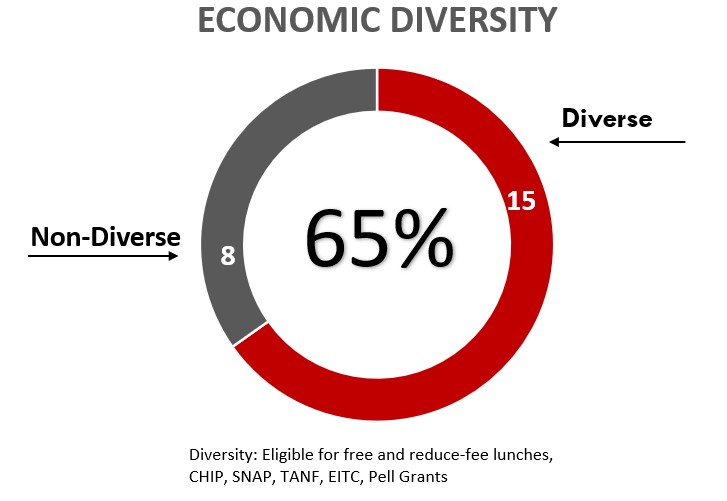

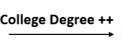

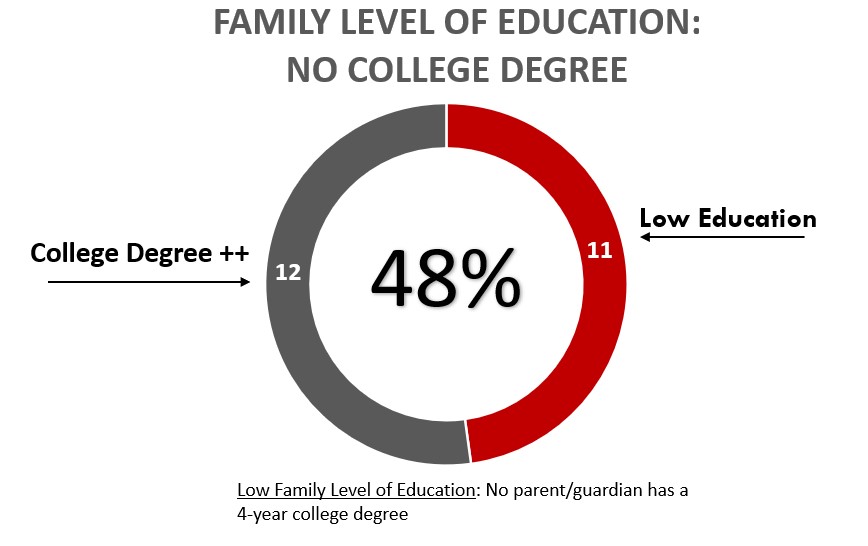


**OTHER LIFE EXPERIENCES**

**
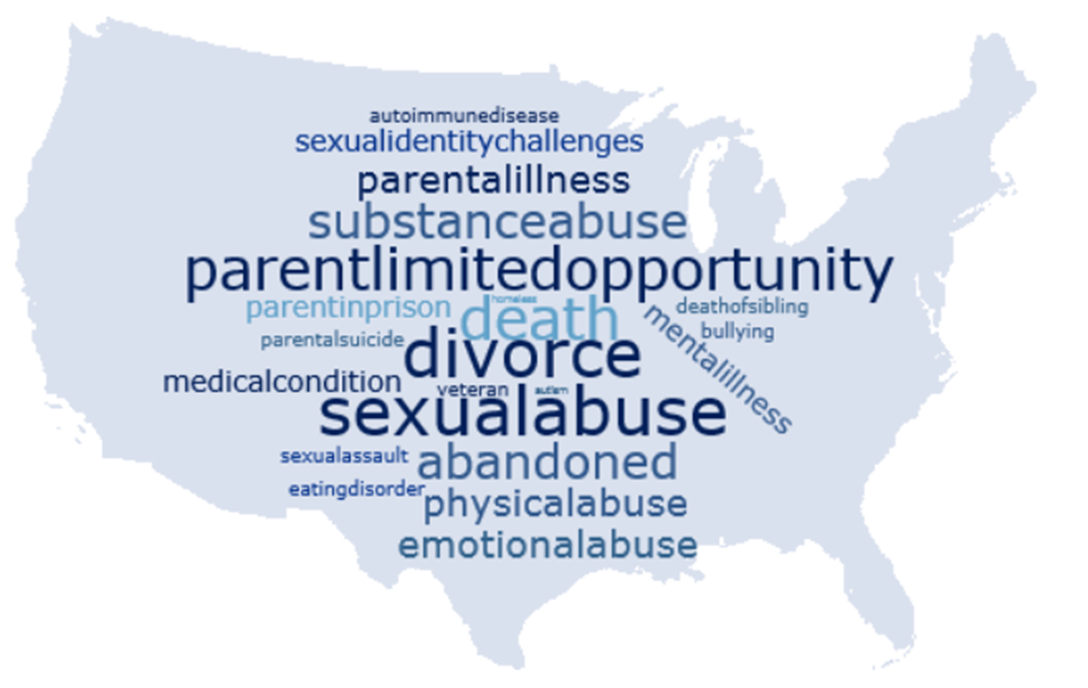
**

25% of Aspirnaut participants over the past 15 years have an Adverse Childhood Experiences (ACE) score of 4 or higher.

1. **Outcomes**

The Aspirnaut holistic approach ensured the success of students in the research lab and propelled them to admission to fifteen universities. The experience provided students with the tools and empowerment to effect positive change in themselves, their families, and communities for generations to come. Their research discoveries revealed chloride pressure is a primordial innovation that drives and maintains the assembly of collagen IV scaffolds.


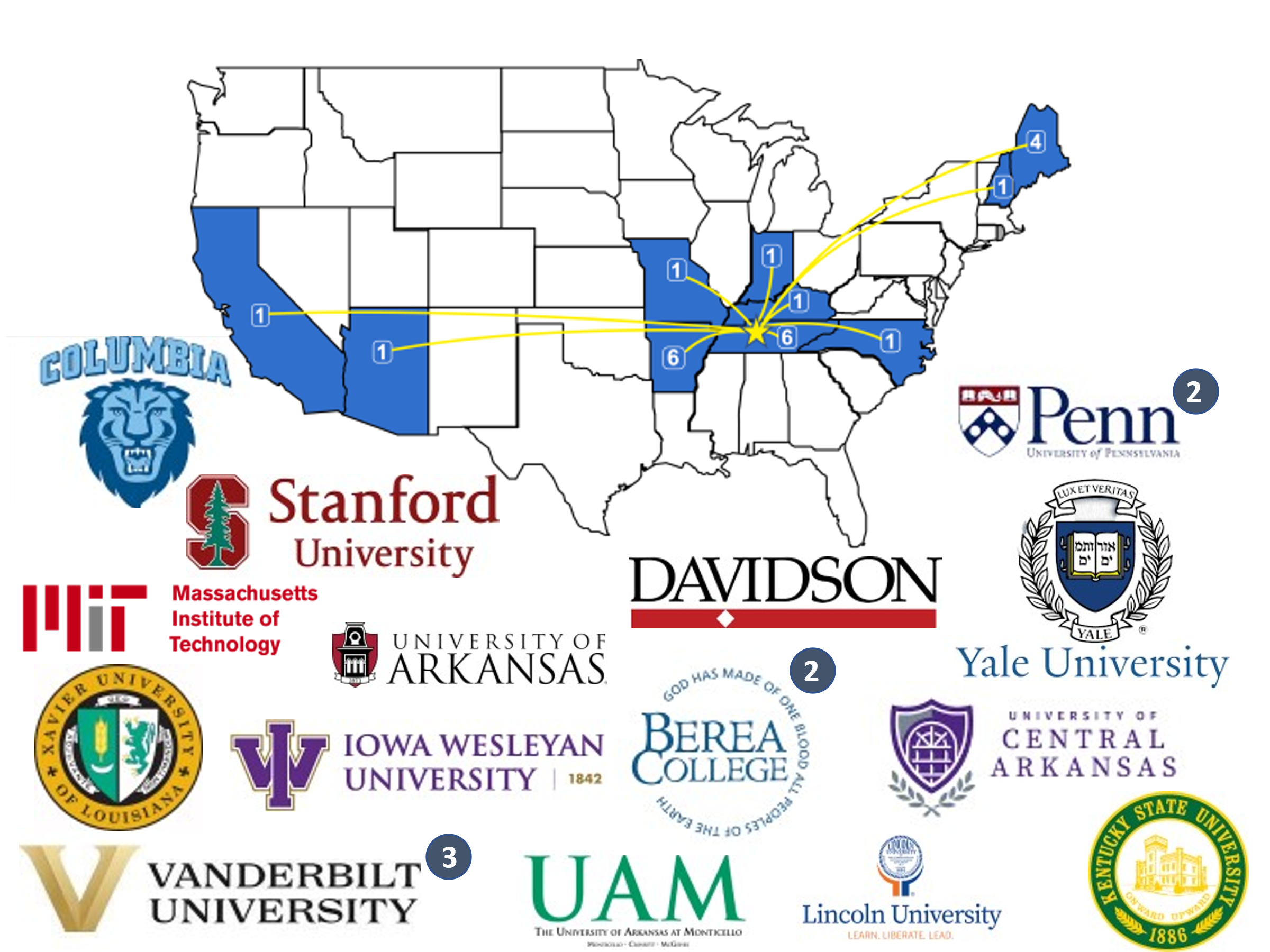


1.
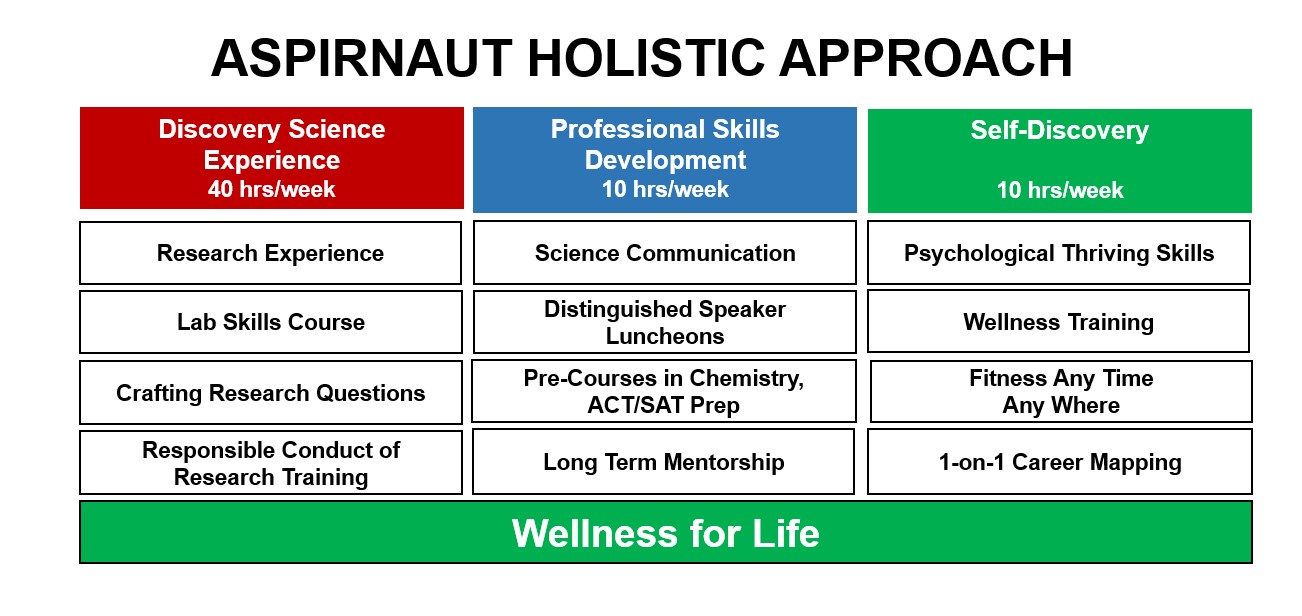
**The Aspirnaut Holistic Approach for Training**

**Recruitment of participants** from rural and diverse backgrounds is key to achieving the Aspirnaut mission. Rural and underserved students are a vital and untapped talent pool for increasing the number and diversity of students entering the STEM workforce. Program awareness is through a variety of online resources and strategic partnerships. Students apply to the program through a website link. Selection focuses on finding talented rural, economically disadvantaged, first-generation college, and ethnically/racially diverse students.

**Guided Discovery Science** includes exploring, generating and interpreting data, and communicating findings in oral and written formats^6^. Crafting research questions is a crucial component of scientific research and commonly utilizes collaboration and debate to enhance learning and broaden the student's scientific knowledge. Debate has been shown to increase critical thinking and research skills and can increase confidence within a subject^.^ A lab skills course is taught during the summer program's first two-and-a-half days to familiarize the participants with the laboratory environment, lab equipment, methodologies, and safety measures. The in-person research lab training and faculty guidance provides a hands-on experience to evaluate proficiency and increase confidence in research skills. Additionally, Aspirnaut partners undergraduate and high school interns, when possible, to help facilitate an environment of teaching and near-peer learning.

**Guided Professional Discovery** aids the student in developing the wide array of skills needed as a professional and scientist and includes science communication, distinguished speaker luncheons, chemistry pre-courses, ACT/SAT prep courses, one-on-one career mapping, and long-term mentorship. Effective science communication is a critical professional skill to share innovations in the scientific community and the general public. Students attend weekly lunch sessions with prominent scientists in a distinguished speaker series. These interactive sessions highlight educational pathways to successful careers in research and other STEM occupations. Prep courses for the ACT, the SAT, general chemistry, and organic chemistry include twenty hours or more on general concepts, strategies, and problem-solving sessions in preparation for college prep or enrolling in college courses. One-on-one career mapping reviews participants' academic progress and outlines their anticipated college and beyond pathways. Participants are encouraged to stay engaged with program staff, faculty, and mentors. Long-term mentoring can build a student's confidence and increase success in college.

**Guided Self-Discovery** includes curriculum about the domains of wellness, fitness for life, psychological thriving, and social activities. Sixty-eight percent of participants include those from geographic areas with a high incidence of cardiovascular disease, diabetes, and obesity^7-9^. Moreover, one in four participants has a self-reported adverse childhood experience (ACE) score of 4/10 or higher, predisposing them to lifelong increased risk of cardiovascular disease, stroke, cancer, diabetes, asthma, COPD, substance abuse, mental illness, and autoimmune disease^4-5^. Strategies to mitigate long-term health risks are provided as an intervention in weekly sessions. These include classroom wellness sessions designed to help participants build skills associated with increasing resilience, making healthy lifestyle choices, and finding balance in all domains of wellness. Physical fitness training focuses on "Fitness Anytime Anywhere" strength and functional movement sessions. Students engage in sessions twice a week for fifty minutes to help increase stability, endurance, and strength, which are known to reduce injury and stress placed on the body during regular activity. A third component of guided self-discovery included sessions with a clinical psychologist to work on psychological thriving skills. Sessions provide skills to manage life events and challenges and destigmatize help-seeking behaviors. Lastly, social activities assist the participants in community-building and networking. The experience fosters an environment of community with like-minded individuals.

**Wellness for Life Training.** Underpinning the participants' summer training is wellness for life. Physicians and scientists have a high rate of depression and suicide compared to the general public^10-11^. Research has shown that incorporating a wellness program positively affects an individual's physical and mental health and overall wellness^12-15^.

**References**

1. Okrent A, Burk A. Science & Engineering Indicators. *NSF*. [https://ncses.nsf.gov/pubs/nsb20212/stem-pathways-degree-attainment-training-and-occupations](about:blank). Accessed 5/1/2023
2. Buchholz K, Richter F. Infographic: Where students choose stem degrees. *Statista Infographics*. 2023 March 16; <https://www.statista.com/chart/22927/share-and-total-number-of-stem-graduates-by-country/>. Accessed 5/1/2023
3. U.S. Bureau of Labor Statistics. Employment in STEM occupations. *U.S. Bureau of Labor Statistics*. 2022 Sept 8. [https://www.bls.gov/emp/tables/stem-employment.htm](about:blank)l. Accessed 5/1/2023
4. Felitti VJ, Anda RF, Nordenberg D, Williamson DF, Spitz AM, Edwards V, Koss MP, Marks JS. Relationship of childhood abuse and household dysfunction to many of the leading causes of death in adults. The Adverse Childhood Experiences (ACE) Study. *Am J Prev Med*. 1998 May;14(4):245-58. <https://doi.org/10.1016/s0749-3797(98)00017-8>. PMID: 9635069.
5. Anda RF, Felitti VJ, Bremner JD, Walker JD, Whitfield C, Perry BD, Dube SR, Giles WH. The enduring effects of abuse and related adverse experiences in childhood. A convergence of evidence from neurobiology and epidemiology. *Eur Arch Psychiatry Clin Neurosci*. 2006 Apr;256(3):174-86. <https://doi.org/10.1007/s00406-005-0624-4>. Epub 2005 Nov 29. PMID: 16311898; PMCID: PMC3232061
6. Constan Z, Spicer JJ. Maximizing Future Potential in Physics and STEM: Evaluating a summer program through a partnership between science outreach and education research. *Journal of Higher Education Outreach and Engagement*. 2015;19(2):117-136. [https://files.eric.ed.gov/fulltext/EJ1067019.pdf](about:blank)
7. Center for Disease Control and Prevention. Adult Obesity Prevalence Maps. <https://www.cdc.gov/obesity/data/prevalence-maps.html>. Accessed 1/26/2022.
8. Center for Disease Control and Prevention. Heart Disease Facts. <https://www.cdc.gov/heartdisease/facts.html>. Accessed 1/26/2022.
9. Center for Disease Control and Prevention. National Diabetes Statistics Report website. <https://www.cdc.gov/diabetes/data/center/slides.html>. Accessed 1/26/2022
10. Schernhammer ES, Colditz GA. Suicide rates among physicians: a quantitative and gender assessment (meta-analysis). *Am J Psychiatry*. 2004 Dec;161(12):2295-302. <https://doi.org/10.1176/appi.ajp161.12.2295>. PMID: 15569903.
11. Satinsky EN, Kimura T, Kiang MV, Abebe R, Cunningham S, Lee H, Lin X, Liu CH, Rudan I, Sen S, Tomlinson M, Yaver M, Tsai AC. Systematic review and meta-analysis of depression, anxiety, and suicidal ideation among Ph.D. students. *Sci Rep*. 2021 Jul 13;11(1):14370. <https://doi.org/10.1038/s41598-021-93687-7>. PMID: 34257319; PMCID: PMC8277873.
12. Ortiz R, Sibinga EM. The role of mindfulness in reducing the adverse effects of childhood stress and trauma. *Children* (Basel). 2017 Feb 28;4(3):16. <https://doi.org/10.3390/children4030016>. PMID: 28264496; PMCID: PMC5368427.
13. Chandler GE, Roberts SJ, Chiodo L. Resilience intervention for young adults with adverse childhood experiences. *J Am Psychiatr Nurses Assoc*. 2015 Nov-Dec;21(6):406-16. <https://doi.org/10.1177/1078390315620609>. PMID: 26711904.
14. Kubzansky LD, Huffman JC, Boehm JK, Hernandez R, Kim ES, Koga HK, Feig EH, Lloyd-Jones DM, Seligman MEP, Labarthe DR. Positive psychological well-being, and cardiovascular disease: JACC Health Promotion Series. *J Am Coll Cardiol*. 2018 Sep 18;72(12):1382-1396. <https://doi.org/10.1016/j.jacc.2018.07.042>. PMID: 30213332; PMCID: PMC6289282.
15. Clark MM, Bradley KL, Jenkins SM, Mettler EA, Larson BG, Preston HR, Liesinger JT, Werneburg BL, Hagen PT, Harris AM, Riley BA, Olsen KD, Vickers Douglas KS. The effectiveness of wellness coaching for improving quality of life. *Mayo Clin Proc*. 2014 Nov;89(11):1537-44. <https://doi.org/10.1016/j.mayocp.2014.04.028>. Epub 2014 Aug 5. PMID: 25107468.
